# Phenotypic and genetic characterization of an *Avena sativa* L. germplasm collection of diverse origin: implications for food-oat breeding in Chile

**DOI:** 10.3389/fpls.2023.1298591

**Published:** 2023-12-21

**Authors:** Mónica Mathias-Ramwell, Valentina Pavez, Marco Meneses, Feledino Fernández, Adriana Valdés, Iris Lobos, Mariela Silva, Rodolfo Saldaña, Patricio Hinrichsen

**Affiliations:** ^1^Programa de mejoramiento genético de avena, Instituto de Investigaciones Agropecuarias (INIA), Centro Regional de Investigación Carillanca, Temuco, Chile; ^2^Laboratorio de Análisis Genético, Instituto de Investigaciones Agropecuarias, Centro Regional de Investigación La Platina, Santiago, Chile; ^3^Facultad de Recursos Naturales, Universidad Católica de Temuco, Temuco, Chile; ^4^Laboratorio de Espectroscopía Infrarrojo Cercano, Instituto de Investigaciones Agropecuarias, Centro Regional de Investigación Remehue, Osorno, Chile; ^5^Laboratorio de Nutrición Animal y Medio Ambiente, Instituto de Investigaciones Agropecuarias, Centro Regional de Investigación Remehue, Osorno, Chile

**Keywords:** oat breeding, genetic diversity, phenotypic diversity, heritability, SSRs

## Abstract

Oats are known for their nutritional value and also for their beneficial properties on human health, such as the reduction of cholesterol levels and risk of coronary heart disease; they are an important export product for Chile. During the last decade (2010-2022) over 90% of the oat cultivated area in Chile has been covered with *Avena sativa* L. cv. Supernova INIA. This lack of genetic diversity in a context of climate change could limit the long-term possibility of growing oats in Chile. The present study is a phenotypic and genetic analysis of 132 oat cultivars and pure lines of diverse origin that can be considered as potential breeding material. The germplasm was evaluated for 28 traits and analyzed with 14 SSR markers. The effects of genotypes on phenotype were significant over all traits (*P* ≤ 0.05). Most traits exhibited moderate to high broad-sense heritability with exceptions such as yield (H^2^ = 0.27) and hulls staining (H^2^ = 0.32). Significant undesirable correlations between traits were generally of small biological importance, which is auspicious for achieving breeding objectives. Some of the heritability data and correlations provided here have not been previously reported. The overall phenotypic diversity was high (H’ = 0.68 ± 0.18). The germplasm was grouped into three phenotypic clusters, differing in their qualities for breeding. Twenty-six genotypes outperforming Supernova INIA were identified for breeding of conventional food-oats. The genetic diversity of the germplasm was moderate on average (He = 0.58 ± 0.03), varying between 0.32 (AM22) and 0.77 (AME178). Two genetic subpopulations supported by the Structure algorithm exhibited a genetic distance of 0.24, showing low divergence of the germplasm. The diversity and phenotypic values found in this collection of oat genotypes are promising with respect to obtaining genetic gain in the short term in breeding programs. However, the similar genetic diversity, higher phenotypic diversity, and better phenotypic performance of the germplasm created in Chile compared to foreign germplasm suggest that germplasm harboring new genetic diversity will be key to favor yield and quality in new oat cultivars in the long term.

## 1 Introduction

Oats are increasingly gaining popularity due to a rise in awareness among consumers about their nutritional value and health benefits; a compound annual growth rate of 4.38% in consumption in the 2023-2028 period is projected in Latin America (Informe de expertos, 2023). The consumption of oat-based products in specific quantities has positive effects in reducing cholesterol levels, risk of cancer and coronary heart disease, as part of a diet low in cholesterol and saturated fat (FDA, 2023). Oats occupy second place in total area of crops grown in Chile, with around 100,000 ha, and a national average yield ranging between 4.5 and 5 tons per hectare, with production mainly destined for export (ODEPA, 2023).

Chilean oat production in the last decade has been based mostly on one genotype, cv. Supernova INIA, a cultivar created by the New Zealand Institute for Plant and Food Research Limited and registered in Chile in 2010. Although a few locally developed cultivars have been released during the last decades, they have not affected the varietal turnover, since 70 to 90% of the oat area is still occupied by Supernova INIA (De la Fuente, 2022), resulting in crop genetic uniformity. The loss of crop genetic diversity in a given area over a period of time, measured through the decline in cultivar number, represents crop genetic erosion that can result in vulnerability, creating the potential for widespread crop failure (Khoury et al., 2022). This is crucial considering that adverse conditions for cultivation of crops, including biotic and abiotic stresses, are expected to increase with climate change and global warming (Ristaino et al., 2021; Skendžić et al., 2021; FAO, 2022).

The focus of the INIA Chile oats breeding program has been the creation of advanced lines with high yield and stability in diverse environments, adequate industrial grain quality for processing, tolerance to lodging and diseases, among others (Beratto, 2006; Mathias-Ramwell et al., 2016). Knowledge of the genetic and phenotypic diversity of the available germplasm would allow a proper conservation of the germplasm for the future and use in the creation of new cultivars. Broadening the genetic background of a species, improving or at least preserving the most relevant economic traits, is key for the development of environmentally resilient new cultivars (Swarup et al., 2021). Modern oat cultivars have exhibited a narrower gene pool than landraces (Montilla-Bascón et al., 2013; Cieplak et al., 2021). Genetic diversity also varies depending on geographic origin, for example oat germplasm from North and South America showed higher genetic diversity than those from Europe, as European genotypes are closely related to each other (Achleitner et al., 2008).

Understanding the genetic diversity of the breeding germplasm, in combination with the phenotypic variation, correlation and heritability of important traits under selection, has facilitated the design of more effective breeding programs (Nava et al., 2010; Krishna et al., 2013; Boczkowska et al., 2016; Winkler et al., 2016; Silveira et al., 2020; Cieplak et al., 2021). There is published research on this aspect in Sweden, Denmark, Finland, Norway (Nersting et al., 2006), USA (Winkler et al., 2016), India (Chauhan and Singh, 2019), and Brazil (Mazurkievicz et al., 2019; Meira et al., 2019; Zimmer et al., 2019; Silveira et al., 2020), but not under the environmental conditions of southern Chile. Few Chilean oat genotypes have been included in genetic diversity studies (Achleitner et al., 2008), which do not include the newest breeding lines and those most cultivated in Chile.

Different types of molecular markers have been used in the genetic characterization of oats, including, including Amplified Fragment Length Polymorphism-AFLP (Achleitner et al., 2008), microsatellites or Simple Sequence Repeats-SSR (Li et al., 2000; Wight et al., 2010; Tanhuanpää et al., 2012), and Single Nucleotide Polymorphism-SNP (Tinker et al., 2014; Zimmer et al., 2019), among others. Despite the rapid development of new molecular tools, SSRs continue to be used, and are considered suitable for genetic diversity studies, and for the inference of population structure (Rana et al., 2019; Raza et al., 2020; Arora et al., 2021). SSRs are also effective in detecting heterogeneity in oat varieties, purity of seed lots, and genetic mapping and fingerprinting studies (Wight et al., 2010; Zheng et al., 2019). SSRs are not affected by external and internal environments, are cost-effective, fast, accurate, simple, highly polymorphic, reliable, and their co-dominant inheritance allow them to distinguish between homozygous and heterozygous loci (Jannink and Gardner, 2005; Zheng et al., 2019; Kaur et al., 2021).

For these reasons, the present study focusses on the phenotypic and SSR-based genetic analysis of 132 oat genotypes of diverse origin, including historical and current advanced pure lines and cultivars. The assessment of the phenotypic and genetic diversity, with other important parameters for the estimation of genetic gain, were addressed with the purpose of understanding the possible causes of the low rate of cultivar turnover in Chile, as well as the status of the available germplasm regarding the breeding goals in the selection of conventional food-oats.

## 2 Materials and methods

### 2.1 Plant material

A collection of 132 oat genotypes of diverse origin representing 85 pure lines, 46 cultivars, and one land race, including historical and modern germplasm, was selected for the study (**Supplementary Table S2**). Seeds of foreign germplasm were provided by the Quaker International Oat Nursery-QION and International Oat Nursery-ION collaborations, and other breeding programs through specific Material Transfer Agreements. The seeds of cultivars and lines created and/or registered in Chile were obtained from the INIA breeding program. The geographic origins of introduced lines were obtained from the POOL online oat database (Tinker and Deyl, 2005).

### 2.2 Field trial and experimental design

A field trial including the 132 oat genotypes was sown on June 15, 2020, in Vilcún (38°41′25′′S, 72°23′32′′ W, La Araucanía Region, Chile). The experimental design was an alpha-lattice with two replications. The experimental unit was a 2 m long and 0.6 m wide plot, resulting in a total of 264 experimental plots. The experiment was arranged in 12 incomplete blocks, each block containing 11 experimental units each corresponding to a different oat genotype. The seeds were disinfected using 2 mg Benomyl (Polyben 50 WP, Anasac S.A., Chile) per g of seeds; the seeding rate was 12 g · m^-2^. The agronomic management consisted of standard control of weeds and fertilizer application, without insecticide or fungicide treatment. The nutrient rates were 14 g · m^-2^ N, using 27% magnesium calcium ammonium nitrate applied 20% at seeding, 40% at early tillering (Zadoks Stage Z-21), and 40% during full tillering (Z-27) (Zadoks et al., 1974); 8 g · m^-2^ P_2_O_5_ applied as monocalcium phosphate at seeding; and 6 g · m^-2^ K_2_O using potassium/magnesium sulfate mixed with monocalcium phosphate at seeding. The herbicide doses were 0.08 mL · m^-2^ of a mix of Flufenacet, Flurtamona, and Diflufenican (Baccara Forte 360 SC, Bayer AG, Leverkusen, Germany) at crop pre-emergence, 0.08 mL · m^-2^ S-metolacloro (Dual Gold 960 EC, Syngenta S.A., Cartagena, Colombia) at early crop emergence, and 0.07 mL · m^-2^ MCPA-dimethylammonium (MCPA 750 SL, A.H. Marks Co., West Yorkshire, England) at full tillage.

### 2.3 Phenotypic trait measurements

A total of 28 phenotypic traits were evaluated in each experimental unit of the field trial (**Supplementary Table S1**). Phenotypic traits were selected based on USDA online Triticeae Toolbox Oat - T3 Oat (https://oat.triticeaetoolbox.org/search/traits), being mostly quantitative traits of importance for breeding. Visual scores of diseases and agronomic types were assessed during tillage, panicle emission, dough and mature grain stages. Plant height and panicle length were measured with a ruler at maturity; lodging was evaluated one day before harvest. After harvest, the grain yield was normalized to 12% moisture. Subsequently, a clean representative sample of 250 g of each experimental unit was obtained for evaluation of quality traits in hulled and dehulled grains.

### 2.4 DNA isolation and SSRs analysis

Ten seeds of each oat genotype were germinated and grown in Petri dishes containing paper towel at room temperature, being periodically moistened with distilled water. Then, 10 mg of fresh leaf tissue were collected from each of the 10 seedlings and pooled, resulting in a total of 100 mg fresh sample per oat genotype. Each pooled sample was fully pulverized using liquid nitrogen. DNA isolation was conducted following a CTAB DNA isolation procedure (Fulton et al., 1995). In previous work conducted in our laboratory in 2014, we had selected 107 SSRs reported in published literature based on their reported quality. Primer pairs for the selected SSRs were then synthesized by Integrated DNA Technologies Inc., USA. A group of 38 SSRs were subsequently pre-selected based on their reproducibility and quality tested against several oat genotypes available at the time under our local laboratory conditions (unpublished work). In the present study, the 38 SSRs previously selected were screened against a subsample of 10 oat genotypes out of the 132 total genotypes studied herein. The selected genotypes were representative of different pedigrees, geographic origins, and included modern and historic cultivars, and pure lines. Out of the 38 SSRs tested, 14 SSRs were selected for the present evaluation of 132 oat genotypes based on their polymorphism and visual quality of the amplification patterns. The polymerase chain reactions (PCR) were conducted in a final volume of 12 µL, using 20 ng DNA, 1X PCR buffer, 0.125 mM dNTPs, 1.5 mM MgCl_2_, 2.5 mM of each primer and 1 U Taq polymerase. The PCR program included a first denaturing step of 94°C for 7 min, 40 cycles of 95°C for 1 min, 54-58°C for 45 sec and 72°C for 1 min, followed by a final elongation at 72°C for 7 min. The amplified fragments were separated in 6% polyacrylamide sequencing gels, at 80 W for 2 to 3 hours, depending on the marker. The fragments were visualized with silver nitrate stain (Narváez et al., 2001).

### 2.5 Phenotypic data analysis

The variance analysis of the phenotypic data was carried out using the model $(Y_{ijk}=\text{µ}+Gen_{i}+Block\left(Rep_{j})+{\text{ε}}_{ijk}\right)$ , which was fitted by applying a restricted maximum likelihood- REML, using the R 4.1.0 software (R Core Team, 2022) along with the Metan package (Olivoto and Lúcio, 2020). The genotype was treated as a random effect to estimate the broad-sense heritability (H^2^) of the 28 phenotypic traits, and the Best Linear Unbiased Predictor-BLUP of the 132 oat genotypes. A multi-trait genotype-ideotype distance index (MGIDI) was calculated for each genotype, using 28 phenotypic traits with their respective breeding objectives (increase, decrease) (**Supplementary Table S1**) (Olivoto and Nardino, 2021).

The linear associations between traits were examined by Pearson correlations, using the adjusted BLUP means obtained through the model. To identify the linear effect between the traits, controlling statistically the effect of others traits, a Pearson correlation matrix was used to calculate partial correlations (Olivoto and Lúcio, 2020). The significance of 465 pairwise trait-trait combinations was tested with the Student’s t (*P* ≤ 0.05). The correlation coefficients were ranked as negligible (│r│< 0.10), weak (0.10 ≥ │r│ ≤ 0.39), moderate (0.40 ≥ │r│ ≤ 0.69), strong (0.70 ≥ │r│ ≤ 0.89), and very strong (│r│ ≥ 0.90) (Mukaka, 2012).

The phenotypic diversity of each trait was estimated with the Shannon-Weaver diversity index (H) (Perry and McIntosh, 1991), using the formula: $\text{H}=\;-\;\sum_{i=1}^{n}P_{i}\;log_{2}\;P_{i}$ , where n is the number of classes of traits and *P_i_* is the proportion of accessions in the *_ith_* class of a trait. Each H value was normalized by dividing it by its maximum value (log2n), which ensured that all values were in the range of 0 to 1 for comparison purposes, called H’ (Perry and McIntosh, 1991). Since the traits were mainly quantitative, the BLUP data were previously scaled to percentages and divided into ten different phenotypic classes ranging from 0 to 100%. The diversity index was categorized as high (H’ ≥ 0.60), intermediate (0.40 ≥ H’<0.60) or low (H’< 0.40) (Yemataw et al., 2018).

To analyze grouping patterns of the oat germplasm, a principal component analysis – PCA and a cluster analysis using the resulting PC coordinates were conducted using the adjusted BLUP means of the 28 phenotypic traits. The analysis was performed using Factoextra (Kassambara and Mundt, 2020), and FactoMineR (Lê et al., 2008) R packages. A group of six oat genotypes were used as references in figures.

### 2.6 Genetic data analyses

The SSR alleles were scored as presence/absence (0/1) and recorded in a data matrix. The polymorphism information content (PIC) was calculated for each marker with the formula: $\text{PIC}=1-\sum_{i=1}^{n}Pi^{2}$ , where n is the number of alleles, and Pi is the frequency of allele i (Weir, 1996). The discriminating power (D) of each marker was estimated with the online iMEC software (Amiryousefi et al., 2018). A genetic distance tree was generated with the Jaccard dissimilarity index, and the unweighted neighbor joining clustering method with 10,000 bootstrap reps, using Darwin 6.0.21 (software available in https://darwin.cirad.fr/).

The genetic structure of the oat germplasm was inferred with the *Structure 2.3.4* software (Pritchard et al., 2000). The simulations assumed K values from 1 to 10 populations, 100,000 burn-in run iterations, 100,000 Markov Chain Monte Carlo, with 10 runs for each K value. The optimal number of populations was estimated observing the Delta K decay with each K value (Evanno et al., 2005). Then the Shannon Information diversity Index (I), Nei’s genetic diversity index (He), observed heterozygosity (Ho), number of alleles, and private alleles, were calculated using GeneAlex v6 (Peakall and Smouse, 2006). Low frequency alleles were pooled to fulfill the data format of GeneAlex. A molecular variance analysis (AMOVA) was also carried out using GeneAlex.

Finally, the phenotypic and genetic diversity indexes, and the MGIDI selection index were calculated in the phenotypic clusters and genetic pools inferred, and in categorical groups formed by origin of the germplasm. Then Kruskal-Wallis non-parametric comparisons were carried out between the groups, with *P* ≤ 0.05 declared as significance and 0.05< *P* ≤ 0.10 considered as a tendency.

## 3 Results

### 3.1 Phenotypic traits variation and heritability

The effect of genotype on phenotype was significant over all traits (*P* ≤ 0.05), showing variation in the studied oat germplasm in all the traits (**Table 1**). The genetic coefficient of variation (CVg) ranged from 0.22% (dry matter content) to 160% (lodging percentage); it was low for grain quality traits such as hectoliter weight (4.16%), groat content (5.65%), dry matter (0.22%) and heading days (3.41%) (**Table 1**). High CVg were found in the incidence of diseases such as *Pseudomonas syringae* (76.96%) and Barley Yellow Dwarf Virus (98.68%), and in the low severity (66.21%) and high severity (62.97%) groat staining (**Table 1**). The lodging percentage was the only trait exhibiting CVg above 100%, showing the high variation in the data (0.4% - 0.94%) in relation to the mean (15.39%) (result not shown). This is likely due to the field trial having low lodging except for a group of sensitive genotypes exhibiting severe lodging (**Supplementary Table S2**).

**Table 1:** Effect of the genotype on phenotypic traits and genetic parameters.

Traits	Variance				*P*	H^2^	CVg
	Genetic	Rep:block	Residual	Phenotypic	value		(%)
Agronomic							
Grain yield	13,754	9,005	28,638	51,397	< 0.001	0.27	13.74
Heading days	29.00	0.15	2.60	31.76	< 0.001	0.91	3.41
Lodging percentage	613.10	5.45	146.48	765.10	< 0.001	0.80	160.90
Lodging severity	0.40	0.02	0.60	1.08	< 0.001	0.43	33.42
Panicle length	6.60	0.00	3.01	9.67	< 0.001	0.69	12.26
Plant height at tillering	8.00	9.16	34.55	51.81	0.048	0.16	4.22
Plant height at maturity	151.10	33.55	33.57	218.30	< 0.001	0.69	9.60
Plant types							
Vigor score	0.05	0.03	0.19	0.26	0.021	0.19	7.99
Agronomic score at tillering	0.17	0.06	0.27	0.50	< 0.001	0.34	6.39
Agronomic score at dough grain	0.94	0.03	0.45	1.42	< 0.001	0.66	15.35
Agronomic score at maturity	0.82	0.00	0.33	1.15	< 0.001	0.71	14.36
Hull color	0.70	0.00	0.13	0.83	< 0.001	0.84	50.79
Panicle type	0.47	0.00	0.01	0.48	< 0.001	0.98	29.03
Incidence of diseases							
Barley Yellow Dwarf Virus	0.15	0.04	0.28	0.48	< 0.001	0.31	98.68
*Drechslera avenae*	187.75	26.01	148.91	362.67	< 0.001	0.52	53.71
*Pseudomonas syringae*	7.11	1.47	22.61	31.20	0.007	0.23	76.96
Grain quality							
Hectoliter weight	5.14	0.20	0.90	6.24	< 0.001	0.82	4.16
Groat content	14.79	0.50	3.06	18.34	< 0.001	0.81	5.65
Broken groats after peeling	12.35	0.00	3.00	15.35	< 0.001	0.80	38.99
Hulled grains after peeling	0.80	0.00	0.50	1.29	< 0.001	0.61	55.41
Hull staining	0.10	0.02	0.20	0.32	< 0.001	0.32	22.91
Low severity groat staining	17.54	0.56	4.57	22.66	< 0.001	0.77	66.21
High severity groat staining	0.44	0.01	0.38	0.83	< 0.001	0.53	62.97
Thousand hulled grain weight	14.75	1.17	2.66	18.58	< 0.001	0.79	8.53
Thousand dehulled grain weight	11.27	0.39	1.26	12.92	< 0.001	0.87	10.51
Groat protein	3.10	0.46	0.44	3.99	< 0.001	0.78	11.40
Groat fat	0.52	0.01	0.13	0.66	< 0.001	0.78	9.11
Groat dry matter	0.04	0.02	0.02	0.08	< 0.001	0.51	0.22

Rep, replicate; H^2^, broad sense heritability; CVg, genetic coefficient of variation.

The contribution of genetic variance to the phenotypic variance ranged from 15.62% (plant height at tillering) to 98.42% (panicle type), resulting in broad sense heritability (H^2^) ranging from 0.16 to 0.98 (**Table 1**). The H^2^ was low for grain yield (0.27) and traits measured at early stages of development in the field, such as vigor score (0.19), agronomic score at tillering (0.34), plant height at tillering (0.16) and *P. syringae* incidence (0.23), showing a major proportion of environmental factors explaining the phenotypic variance. In contrast, high H^2^ was observed for grain quality traits such as hectoliter weight (0.82), groat content (0.81), groat protein (0.78) and fat (0.78) content, and morphological traits like panicle type (0.98) and hull color (0.84).

### 3.2 Phenotypic trait diversity

To identify traits as potential breeding targets in the studied germplasm, the phenotypic diversity was estimated based on the Shannon-Weaver diversity index (H). H was normalized by its maximum number of classes, obtaining H’ values in the range between 0 and 1 for comparative purposes (Perry and McIntosh, 1991). Thus H’ allowed us to classify the traits in different diversity categories, excluding the effect of the number of classes. The H index, reflecting the abundance of oat genotypes in different phenotypic classes, was 1.71 ± 0.69, ranging from 0.45 (hectoliter weight) to 2.63 (*Drechslera avenae* incidence); whereas H’ was 0.68 ± 0.18, ranging between 0.28 (hectoliter weight) and 0.94 (lodging severity) (**Table 2**). Twenty-four of 28 traits were in the high (H’ > 0.6), four traits in the intermediate (0.40 ≥ H’< 0.60) and two traits in the low (H’< 0.40) diversity categories.

**Table 2:** Frequency of the germplasm in different phenotypic classes and phenotypic diversity.

Traits	BLUPs range		Frequency at each phenotypic class (%)										Diversity	
	Min	Max	1	2	3	4	5	6	7	8	9	10	H	H’
Agronomic														
Grain yield, g · m^-2^	596.5	1,035.4	–	–	–	–	–	2	6	24	55	14	1.70	0.73
Heading days, d	138.4	177.9	–	–	–	–	–	–	–	2	60	38	1.10	0.69
Lodging percentage, %	0.4	91.4	67	11	2	6	2	2	1	2	3	4	1.80	0.54
Lodging severity, 1-5 rating	1.3	3.1	–	–	–	–	30	13	15	12	23	7	2.43	0.94
Panicle length, cm	15.7	29.9	–	–	–	–	–	8	40	44	5	2	1.70	0.73
Plant height at tillering, cm	64.4	73.4	–	–	–	–	–	–	–	–	16	84	0.63	0.63
Plant height at maturity, cm	97.2	166.3	–	–	–	–	–	2	12	58	24	5	1.62	0.70
Plant types														
Vigor score, 1-4 rating	2.2	3.0	–	–	–	–	–	–	–	2	30	68	0.99	0.62
Agronomic score at tillering, 1-10 rating	5.1	6.9	–	–	–	–	–	–	–	2	23	76	0.88	0.56
Agronomic Score at dough grain, 1-10 rating	2.8	7.7	–	–	–	1	2	3	7	30	39	20	2.07	0.74
Agronomic score at maturity, 1-10 rating	3.5	7.7	–	–	–	–	2	5	7	29	45	14	1.99	0.77
Hull color, 0-5 rating	0.1	4.7	1	–	50	5	30	2	6	1	3	2	1.97	0.62
Panicle type, 1-3 rating	1.0	2.9	–	–	–	12	–	–	39	–	2	47	1.50	0.75
Incidence of diseases														
Barley Yellow Dwarf Virus, %	0.0	1.6	12	41	23	8	9	5	1	1	1	1	2.40	0.72
*Drechslera avenae*, %	7.4	60.4	–	6	22	20	24	13	2	6	5	2	2.73	0.86
*Pseudomonas syringae*, %	1.7	11.4	–	30	33	21	8	4	2	2	–	1	2.28	0.76
Grain quality														
Hectoliter weight, kg · hL^-1^	45.9	64.5	–	–	–	–	–	–	–	8	92	1	0.45	0.28
Groat content, %	53.6	96.5	–	–	–	–	–	1	43	55	–	1	1.10	0.55
Broken groats after peeling, %	1.1	23.5	1	3	23	36	24	6	4	1	1	2	2.34	0.70
Hulled grains after peeling, %	0.3	4.9	1	17	41	20	10	5	2	2	2	1	2.39	0.72
Hull staining, 0-3 rating	0.7	1.9	–	–	–	1	–	8	37	33	16	6	2.06	0.80
Low severity groat staining, %	1.3	20.7	3	32	26	20	5	5	4	2	3	1	2.55	0.77
High severity groat staining, %	0.3	3.5	2	31	27	19	9	7	4	1	–	1	2.45	0.77
Thousand hulled grain weight, g	28.1	55.3	–	–	–	–	–	1	3	36	55	5	1.41	0.61
Thousand dehulled grain weight, g	23.4	39.5	–	–	–	–	–	1	11	31	43	14	1.85	0.80
Groat protein, %	11.7	21.3	–	–	–	–	–	5	40	39	15	2	1.77	0.76
Groat fat, %	6.0	9.3	–	–	–	–	–	–	2	26	45	27	1.65	0.83
Groat dry matter, %	89.9	90.9	–	–	–	–	–	–	–	–	–	100	0.00	0.00
Overall													1.71	0.68
SD													0.69	0.18

BLUPs, best linear unbiased predictors; H, Shannon Weaver diversity index; H’, H scaled by the number of phenotypic classes. The phenotypic diversity was categorized as high (H’ ≥ 0.60), intermediate (0.40 ≥ H’< 0.60) or low (H’< 0.40). The underlined numbers indicate the classes of the reference cultivar Supernova INIA.

Grain yield had high diversity (H’ = 0.73). However, only 14% of the oat genotypes outperformed Supernova INIA (**Table 2**). Most of the genotypes were in the high phenotypic classes for grain quality traits such as hectoliter weight, groat content and thousand hulled grain weight, but a few genotypes had higher quality than Supernova INIA. A similar pattern was observed for heading days with most genotypes exhibiting intermediate to long cycles, but 2% of genotypes emitted their panicles earlier than Supernova INIA. Plant height at maturity exhibited high diversity (H’ = 0.70), with a high proportion of intermediate to tall genotypes.

Groat protein (H’ = 0.76) and fat (H’ = 0.83) content showed high diversity, with a similar fraction of genotypes with higher and lower values than Supernova INIA (**Table 2**). High diversity was also found for grain quality traits including hulled groats and broken grains after peeling, high and low severity grain staining, hull staining, and foliar diseases; a good proportion of genotypes had better quality and tolerance to diseases than Supernova INIA. All oat genotypes were assigned to the same phenotypic class for groat dry matter content, resulting in null diversity.

### 3.3 Pairwise correlations between traits

**Figure 1:**
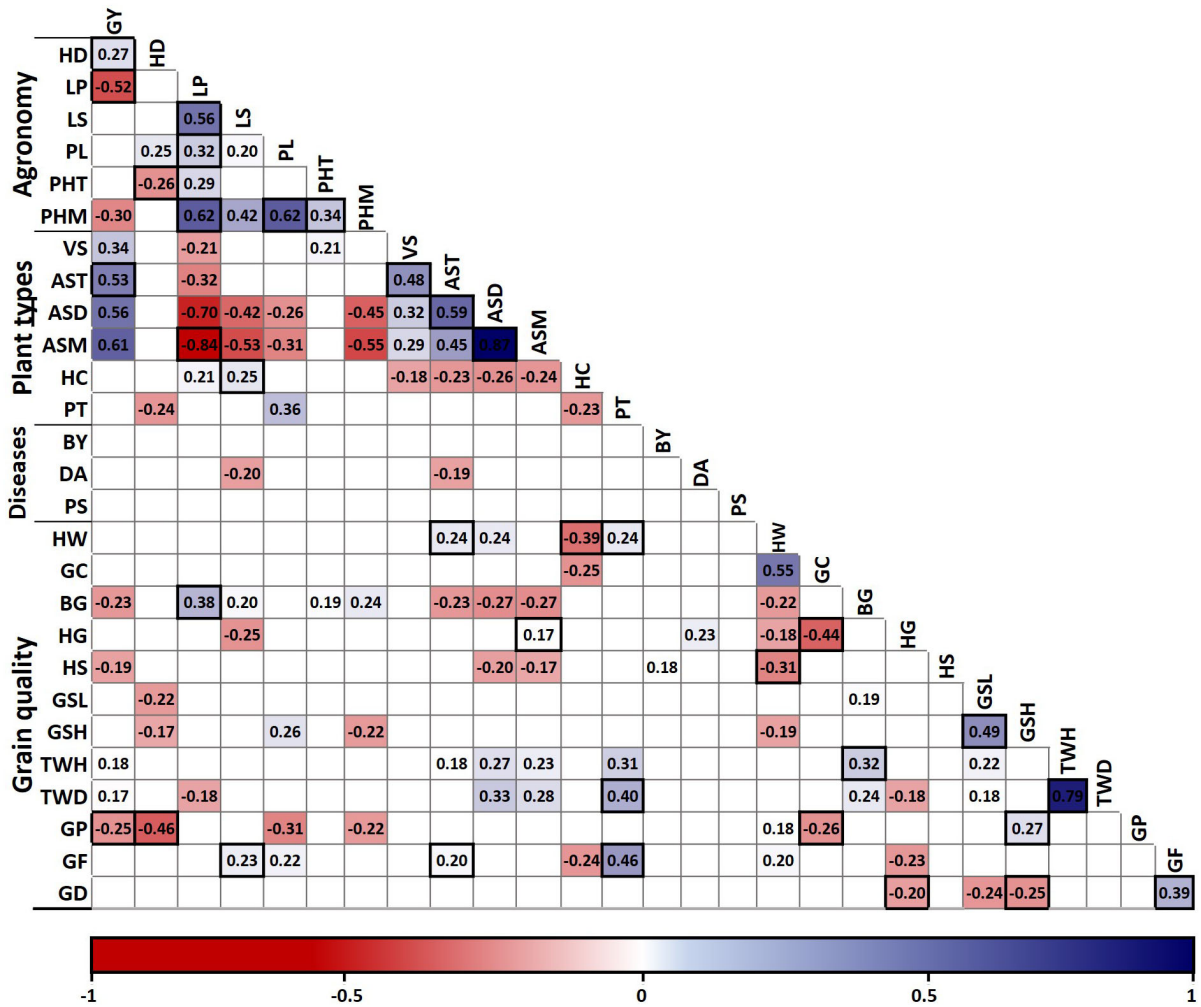
Pearson correlation matrix of 28 traits measured in 132 oat genotypes and partial correlation validation. Only correlations with *P* ≤ 0.05 are shown in the graph. Significant associations in the partial correlation analysis are marked with black boxes. GY, grain yield; HD, heading days; LP, lodging percentage; LS, lodging severity; PL, panicle length; PHT, height at tillering; PHM, height at maturity; VS, vigor score; AST, agronomic score at tillering; ASD, dough grain; and ASM, maturity; HC, hull color; PT, panicle type; BY, barley yellow dwarf virus incidence; DA, *Drechslera avenae* incidence; PS, *Pseudomonas syringae* incidence; HW, hectoliter weight; GC, groat content; BG, broken groats and HG, hulled grains after peeling; HS, hull staining; GSL, low severity and GSH, high severity groat staining; TWH, thousand hulled and TWD, thousand dehulled grain weight; GP, groat protein; GF, groat fat; and GD, groat dry matter content.

Since Pearson´s correlation does not consider the influence of other traits on the relationship between two traits, a partial correlation analysis was used to control statistically the effect of other traits on the correlations (Olivoto and Lúcio, 2020). Forty-four associations were significant both with Pearson and partial correlation analysis, representing robust associations (**Figure 1**). However, 64 associations were significant only with Pearson correlation, whereas 18 associations were found significant only in the partial correlation analysis (**Supplementary Table S3**).

As expected, the association between related traits such as thousand hulled and dehulled grain weight and agronomic scores at dough and mature grain stages was strong, whereas the associations between the other traits were mostly weak to moderate (**Figure 1**). Grain yield exhibited positive associations with the agronomic scores at tillering and maturity, and negative associations with lodging percentage, plant height at maturity and groat protein content. Therefore, grain yield associations were mostly favorable for genetic breeding, except for groat protein. Also, plant height at maturity exhibited a positive association with lodging percentage, plant height at tillering and panicle length.

Hectoliter weight showed a positive association with groat content and panicle type, and negative associations with hull color and hull staining; all these associations are favorable for genetic breeding of high grain quality oats (**Figure 1**). As expected, high and low severity groat staining exhibited a positive association. However, high severity groat staining had a positive association with protein content and negative with groat dry matter content, whereas low severity groat staining exhibited a negative association with heading days. Also, groat protein had a negative association with heading days and groat content.

### 3.4 Phenotypic principal component analysis and clustering of the oat germplasm

A multivariate analysis was performed on the 132 oat genotypes values, to reduce complexity and explore the relationships among several traits of economic importance. Dimension 1 (17.41%) was mainly represented by agronomic plant type scores and grain yield, while Dimension 2 (11.09%) was associated with grain quality traits (**Supplementary Figures S1A, B**). The genotypes resulted in a mostly well distributed germplasm but with a group of nine genotypes markedly separated from the others (**Supplementary Figure S1C**).

**Figure 2:**
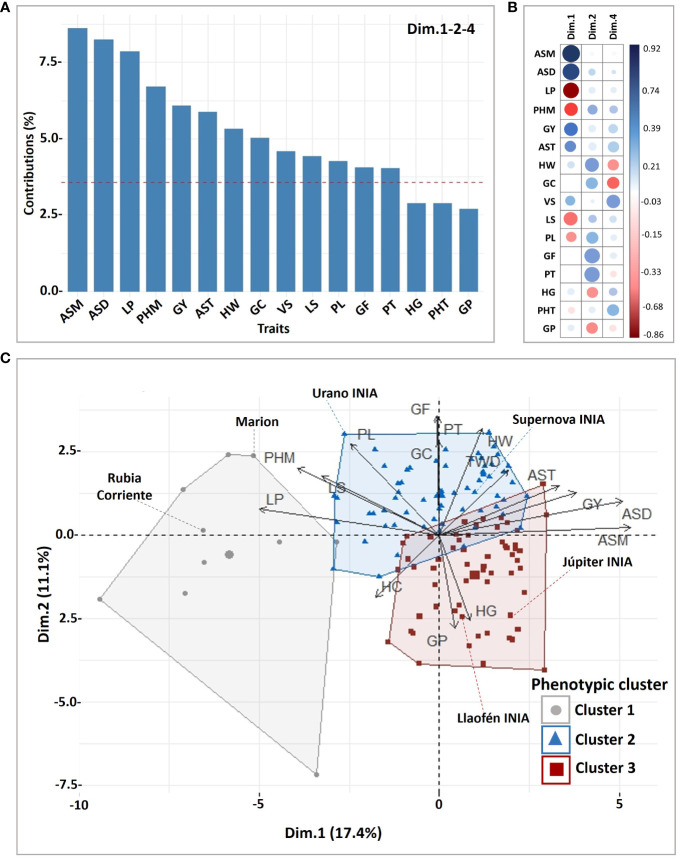
Phenotypic cluster analysis for 28 traits and 132 oat genotypes. **(A)** Contribution of the main traits explaining the variance of significant (*P* ≤ 0.05) dimensions in the cluster analysis, **(B)** correlation of the traits and the dimensions, **(C)** biplot of genotypes and traits showing the resulting clusters. GY, grain yield; LP, lodging percentage; LS, lodging severity; PL, panicle length; PHT, height at tillering; PHM, height at maturity; VS, vigor score; AST, agronomic score at tillering; ASD, dough grain; and ASM, maturity; PT, panicle type; HW, hectoliter weight; GC, groat content and GF, groat fat; HG, hulled grains; GP, groat protein.

The grouping analysis of the principal component coordinates found significant explanation of the clusters mainly in 13 traits linked to the first, second and fourth PCA dimensions, representing 35.17% of the explained variance (**Figure 2A**; **Supplementary Table S4**). Depending on the correlations of the traits in relation to the dimensions of the PCA (**Figure 2B**), each cluster exhibited a higher or lower mean compared to the overall mean of the 132 oat genotypes (**Supplementary Table S5**).

The oat genotypes were grouped in three phenotypic clusters, Cluster 1 (N = 10), Cluster 2 (N = 63) and Cluster 3 (N = 59) (**Figure 2C**; **Supplementary Table S5**). Cluster 1 had long panicles but with very high plant height and high lodging, low grain yield, and low industrial grain quality. It was composed mainly of foreign historical cultivars from United States of America (N = 3), Canada (N = 1), Italy (N = 1), Austria (N = 1), Australia (N = 1), Uruguay (N = 1), a pure line from Brazil (**Supplementary Table S2**), and a Chilean landrace (Rubia Corriente), widely used in Chile as forage. Cluster 2 had high grain yield, good industrial grain quality, intermediate plant height, intermediate panicle length, moderate to low groat protein, and slightly high groat fat, such as Supernova INIA and Urano INIA. Cluster 3 genotypes had overall lower plant height, lower lodging, high groat protein, slightly low fat groat contents, slightly higher groat staining, lower industrial grain quality, and shorter panicles, than Cluster 2, for example Júpiter INIA.

### 3.5 Phenotypic value of the oat germplasm for oat-food breeding

To estimate the phenotypic value of the oat germplasm we estimated a Multi-trait Genotype-Ideotype Distance Index (MGIDI) that quantifies each oat genotype with regard to breeding objectives; the lower the index the closer the individual is to the ideotype and therefore higher genetic gain is expected (Olivoto and Nardino, 2021). The MGIDI considered the 28 traits with their respective breeding objectives (increase, decrease), according to the expected attributes in conventional food-oats, such as high field performance and grain quality (**Supplementary Table S1**).

**Figure 3:**
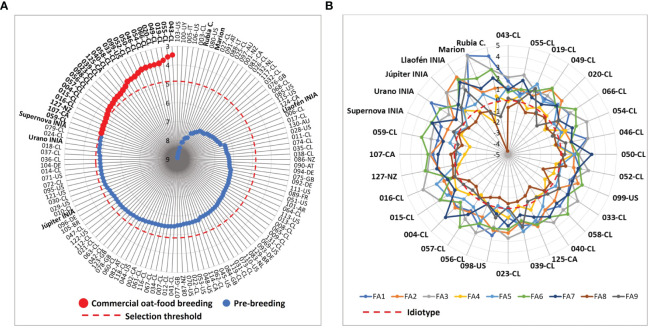
Multi-trait genotype–ideotype distance indexes (MGIDI) of the 132 oat genotypes considering the objectives for conventional food-oat breeding with 28 traits. **(A)** selection of the better germplasm for commercial food-oat breeding and pre-breeding, according to MGIDI scores, **(B)** weaknesses and strengths, corresponding to the qualities for breeding of the selected genotypes and reference cultivars. FA: factors. FA1: grain yield, height at maturity, panicle length, lodging percentage, lodging severity, dough grain and maturity; FA2: thousand hulled and dehulled grains weight, broken grains after peeling; FA3: low severity groat staining, high severity groat staining, *Pseudomonas syringae* incidence; FA4: plant height at tillering, vigor score, agronomic score at tillering; FA5: groat content, hulled grains after peeling, *Drechslera avenae* incidence; FA6: groat protein, heading days; FA7: hull staining, barley yellow dwarf virus incidence; FA8: hectoliter weight, hull color; FA9: groat dry matter, groat fat content, panicle type.

The MGIDI index ranged from 3.46 to 8.91 with a mean of 5.66, while the reference cultivar Supernova INIA had a value of 4.78 (**Figure 3A**, **Supplementary Table S2**). A group of 26 genotypes with better phenotypic performance than Supernova INIA (MGIDI< 4.78) were selected as promising material for food-oat commercial breeding. The group had selection gain in almost all traits, excepting plant height at maturity and groat fat content (**Supplementary Table S6**). The selected genotypes showed different qualities in relation to Supernova INIA (**Figure 3B**); the germplasm was mainly from Chile (N = 21), Canada (N = 2), United States of America (N = 2) and New Zealand (N = 2) (**Supplementary Table S2**). The genotypes ranked in the lower 5% extreme of the MGIDI scores were chosen as potential candidate lines for direct development of new food-oat cultivars. The 105 genotypes with higher MGIDI (lower phenotypic performance) scores than Supernova INIA were kept for pre-breeding purposes, due to their specific characteristics of interest and possibly other potential non-characterized beneficial properties, their conservation being relevant for future studies.

### 3.6 Genetic variation revealed by the SSRs

A total of 64 alleles from 14 SSRs were detected in the 132 oat genotypes. These markers were polymorphic, and 20 alleles exhibited frequencies lower than 0.05 in the set of samples (**Table 3**). The number of alleles per marker ranged from 2 (AME019, AME055, and AME076) to 10 (AME178), with an average of 4.27 alleles per locus; the PIC ranged between 0.26 (AME177) and 0.84 (AME178), with an average of 0.58; and the discriminatory power ranged from 0.28 (MAMA_5) to 0.57 (AME102), with a mean of 0.43. Thus, the polymorphism and discrimination power achieved by this set of SSRs can be considered intermediate. The allele frequencies are detailed in **Supplementary Figure S2**.

**Table 3:** Primer sequences, and efficiency indexes of the SSRs.

SSRs	5´-> 3´Primer sequences	SSR	Ta	Alleles (N)			PIC	D	SSR´s reference
		Type	(°C)	Common	Rare	Total			
				(> 0.05)	(≤ 0.05)				
AM14	F: TGGGTGGCGAAGCGAATC	Genomic	58	4	0	4	0.33	0.44	Li et al. (2000)
	R: GTGGTGGGCACGGTATCA								
AM22	F: AAGAGCGACCCAGTTGTATG	Genomic	56	2	1	3	0.61	0.49	Li et al. (2000)
	R: ATTGTATTTGTAGCCCCAGTTC								
AME013	F: ACGGAACTTCAACACTTTGG	EST	56	2	2	4	0.55	0.38	Tanhuanpää et al. (2012)
	R: GGCATGAGAGTTTTTATGAACC								
AME019	F: CATCACAGTCGCAGCCATG	EST	58	2	0	2	0.56	0.38	Tanhuanpää et al. (2012)
	R: GCATGCATTTTCCCCTCACG								
AME055	F: TTCGACCATGGGAATCTTTG	EST	56	2	0	2	0.57	0.54	Tanhuanpää et al. (2012)
	R: CGGAGGTGCAAACCCTAGTA								
AME076	F: CATGATCCATCACACATACCG	EST	56	2	0	2	0.57	0.57	Tanhuanpää et al. (2012)
	R: CGAATGGATGCTGAATTGG								
AME102	F: GCTGCCTCTACATGAGCAGA	EST	58	4	0	4	0.70	0.57	Tanhuanpää et al. (2012)
	R: TCCTCCTCCAGGATGTGACT								
AME154	F: GTACACACATCCAATCCATTTC	EST	54	3	0	3	0.69	0.46	Tanhuanpää et al. (2012)
	R: TGAAGGAACGGAAATCTGAAG								
AME177	F: ATCGGGTACTAGTGATACATAC	EST	54	3	3	6	0.26	0.54	Tanhuanpää et al. (2012)
	R: CATGTATCTCATCCCAAACTC								
AME178	F: TGTCTTATCTGGCTGGAGCA	EST	56	4	6	10	0.84	0.43	Tanhuanpää et al. (2012)
	R: AGAATTGGAACCGTGTGTAAC								
MAMA_1	F: CATGCTGGCGAAATCTATCA	Genomic	56	5	0	5	0.74	0.47	Wight et al. (2010)
	R: GTGCGCCTCTAACGAAAAAT								
MAMA_5	F: AACCCTAATTACTGCTCCGTTTC	Genomic	56	4	4	8	0.79	0.28	Wight et al. (2010)
	R: GGATTGGGACTTCGCATCTA								
MAMA_11	F: GACTACCGCCCAGATGAGAC	Genomic	56	3	4	7	0.67	0.51	Wight et al. (2010)
	R: TGTATGCACCGATGCAATTT								
MAMA_13	F: CGATGCACTCAGATTTGGAA	Genomic	56	4	0	4	0.79	0.33	Wight et al. (2010)
	R: CTGGATCAAGCAGACATGGA								
Mean				2.80	1.30		0.58	0.43	
Total				44	20				

F, forward; R, reverse; Ta, annealing temperature of primers; PIC, polymorphism information content; D, discriminating power.

**Figure 4:**
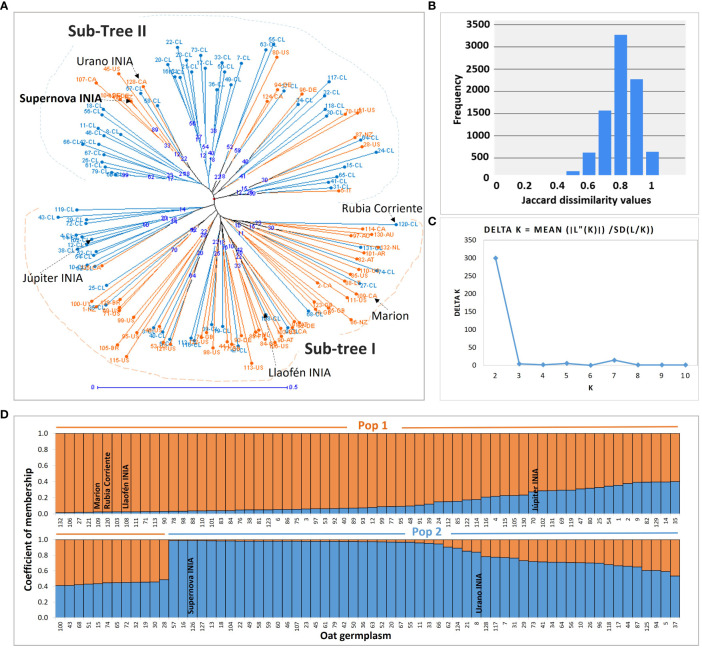
Genetic tree and population structure of the oat germplasm. **(A)** The genotypes created in Chile and the foreign germplasm are marked in light blue and orange, respectively; blue numbers in nodes are the bootstrap support of the branches; sub-tree I, and sub-tree II, are the main groups detected visually. **(B)** Frequency histogram of the Jaccard dissimilarity indexes with 132 oats and 10,000 bootstraps. **(C)** Change in likelihood of the data L(K) at values of K populations from 1 to 10, used to infer the true value of (K) **(D)** Populations inferred with the Structure algorithm. The number labels in **(A, D)** representing the 132 oat genotypes are consistent with **Supplementary Table S2**.

Two main groups were visually different in the neighbor joining genetic tree; sub-tree I grouped mainly Chilean germplasm with a low proportion of introduced lines and cultivars such as Supernova INIA (126-NZ) and Urano INIA (128-CA); sub-tree II grouped mostly foreign germplasm, historical germplasm, and a low proportion of Chilean advanced lines (**Figure 4A**; **Supplementary Table S2**). The genetic dissimilarity between the oats was high, with an average of 0.75, indicating a high degree of genetic differentiation between the oat genotypes, with exception of the Chilean pure lines 59-CL and 60-CL, revealed as duplicates (**Figure 4B**; **Supplementary Table S2**).

### 3.7 Population structure

The Structure analysis with the SSRs supported two subpopulations based on the decay of Delta K, suggesting the existence of two genetic pools in the germplasm (**Figures 4C, D**). The pairwise genetic distance and the similarity between Pop 1 and Pop 2, were 0.24 and 0.79, respectively (**Supplementary Table S7**). The molecular variance analysis found significant fixation indexes, indicating significant genetic variation among subpopulations (F_ST_), among individuals (F_IS_), and within individuals (F_IT_) (*P* = 0.001), representing the 11%, 77%, and 12% of the molecular variance, respectively (**Supplementary Table S8**).

Fifty-nine percent (N = 78) of the genotypes were assigned to Pop 1, and the other 41% (N = 54) to Pop 2. However, 23.48% of the germplasm was admixed, corresponding to genotypes exhibiting less than 0.7 membership to their respective subpopulation (Wang et al., 2023). The admixed germplasm was higher in Pop 1 (N = 23) than in Pop 2 (N = 8) (**Supplementary Table S2**). Most foreign and historical genotypes, such as Rubia Corriente, Eva, Llaofén INIA, also of the cultivar Júpiter INIA, belonged to Pop 1 (**Supplementary Table S2**). Seventy percent of modern Chilean advanced pure lines, and the most important commercial cultivars in Chile such as Supernova INIA and Urano INIA, were assigned to Pop 2.

The comparison of the genetic distance tree to the population structure showed a similar but not identical grouping of the genotypes; sub-tree I was analogous to Pop 1, and sub-tree II was similar to Pop 2. Six and nine genotypes of sub-tree I and sub-tree- II, respectively, were assessed inversely to Pop 2 and Pop 1 (**Supplementary Table S2**).

### 3.8 Genetic diversity

The overall genetic diversity of the 132 oat genotypes was intermediate (He = 0.58 ± 0.03); diversity was greater in Pop 1 (He = 0.59 ± 0.04) than in Pop 2 (He = 0.44 ± 0.05) (**Table 4**). Extending the analysis per population, the observed alleles per marker ranged between 2 and 9, and between 2 and 5, and the total observed alleles over all markers were 61 and 46, for Pop 1 and Pop 2, respectively. The expected allele number per locus varied from 1.57 to 5.16 in Pop 1, and between 1.05 and 3.26 in Pop 2 (**Table 4**). A total of 14 private alleles were detected in Pop 1 with the SSRs AME102 (N = 1), MAMA_11 (N = 3), AME178 (N = 4), MAMA_1 (N = 1), MAMA_5 (N = 4), AM22 (N = 1); this resulted in 40 oat genotypes containing one to three private alleles (**Supplementary Table S2**). Allele frequencies are provided in **Supplementary Figure S2**.

**Table 4:** Genetic diversity indexes in the inferred subpopulations and in all genotypes.

SSRs	Sub-population 1 (N = 78)					Sub-population 2 (N = 54)					Total (N = 132)				
	Na	Ne	I	Ho	He	Na	Ne	I	Ho	He	Na	Ne	I	Ho	He
AM14	4	2.01	0.95	0.35	0.50	4	3.26	1.25	0.67	0.69	4	2.61	1.15	0.48	0.62
AM22	3	1.81	0.76	0.00	0.45	2	1.05	0.12	0.00	0.05	3	1.47	0.58	0.00	0.32
AME013	4	2.23	0.95	0.04	0.55	4	1.35	0.53	0.00	0.26	4	1.86	0.83	0.03	0.46
AME019	2	1.60	0.56	0.00	0.38	2	1.86	0.65	0.00	0.46	2	1.71	0.61	0.00	0.42
AME055	2	1.54	0.53	0.00	0.35	2	1.26	0.36	0.00	0.21	2	2.00	0.69	0.00	0.50
AME076	2	1.97	0.69	0.00	0.49	2	1.91	0.67	0.00	0.48	2	2.00	0.69	0.00	0.50
AME102	4	3.37	1.29	0.07	0.70	3	1.42	0.57	0.04	0.30	4	3.05	1.22	0.06	0.67
AME154	3	2.60	1.02	0.00	0.61	3	1.83	0.79	0.00	0.45	3	2.27	0.94	0.00	0.56
AME177	6	2.75	1.19	0.74	0.64	5	2.55	1.08	0.72	0.61	6	2.76	1.18	0.73	0.64
AME178	9	5.38	1.89	0.00	0.81	5	2.70	1.14	0.00	0.63	9	4.42	1.72	0.00	0.77
MAMA1	5	2.77	1.29	0.00	0.64	4	2.21	1.03	0.00	0.55	5	2.53	1.22	0.00	0.61
MAMA5	8	4.37	1.71	0.01	0.77	4	2.64	1.09	0.00	0.62	8	3.91	1.64	0.01	0.74
MAMA11	5	2.99	1.23	0.00	0.67	2	1.40	0.46	0.00	0.29	5	2.49	1.07	0.00	0.60
MAMA13	4	3.02	1.22	0.01	0.67	4	2.74	1.17	0.00	0.64	4	3.62	1.32	0.01	0.72
Mean	4.3	2.74	1.09	0.09	0.59	3.3	2.01	0.78	0.10	0.44	4.4	2.62	1.06	0.09	0.58
SD	0.6	0.29	0.11	0.06	0.04	0.3	0.18	0.09	0.07	0.05	0.6	0.23	0.10	0.06	0.03

Na, number of observed alleles; Ne, effective number of alleles; I, Shannon information index. Ho, observed heterozygosity; and He, Nei´s diversity index.

The observed heterozygosity (Ho) was near zero for the majority of SSRs, with exception of AM14 (Ho = 0.48), and AME177 (Ho = 0.73) (**Table 4**). The heterozygosity fluctuated between 0 and 0.33 in the 132 individual oat genotypes, with a mean of 0.09, indicating a low degree of genetic segregation and/or contamination, considering that the DNA was obtained from the combined extraction of 10 seedlings per genotype (**Supplementary Table S2**).

### 3.9 Phenotypic value and diversity of the germplasm by groups

**Figure 5:**
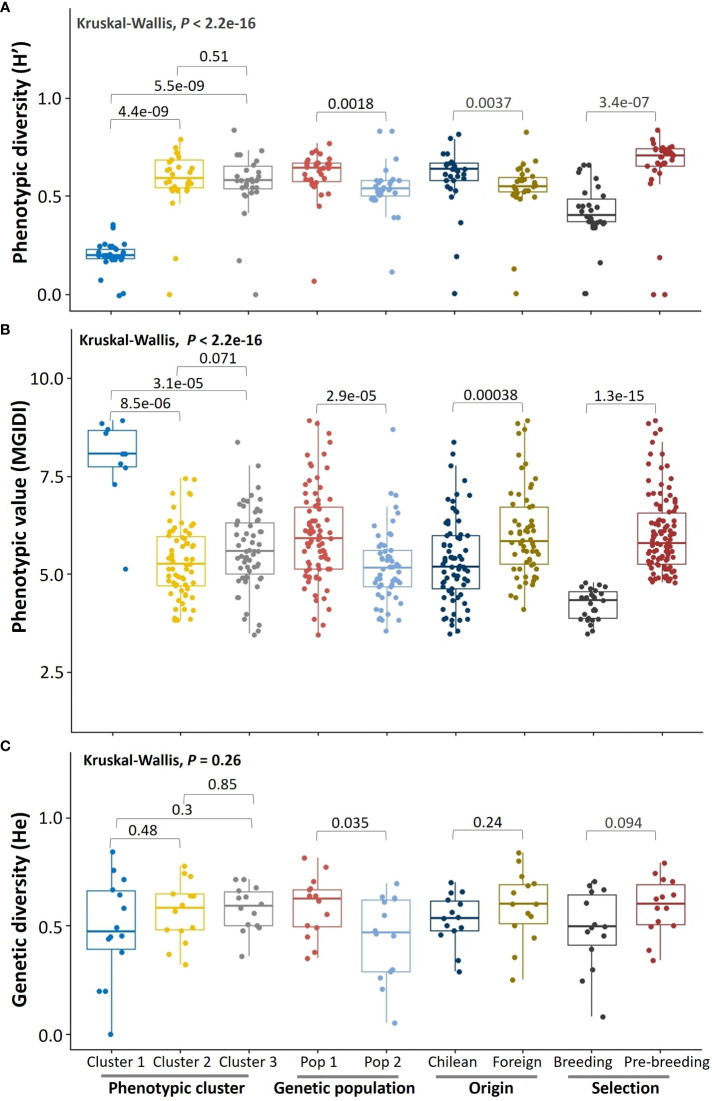
Overall phenotypic performance and diversity of the germplasm by groups. **(A)** Shannon-Weaver scaled phenotypic diversity, **(B)** multi-trait genotype-ideotype index, and **(C)** Nei’s genetic diversity index. The error bars are the minimum and maximum values; the horizontal line in the box is the median. The Chilean germplasm corresponds to genotypes created in Chile using diverse origin germplasm.

To obtain an overview of the germplasm in terms of phenotypic performance and diversity, we applied non-parametric comparisons between phenotypic clusters, genetic populations, in categorical groups by geographic origin (Chilean, foreign), and selection for different uses (commercial food-oat breeding and pre-breeding). The phenotypic diversity (H´) was higher in Cluster 2 (H’= 0.62 ± 00.18) and Cluster 3 (H’ = 0.60 ± 0.17) than in Cluster 1 (H’ = 0.21 ± 0.06), in Pop 1 (H’= 0.63 ± 0.07) than in Pop 2 (H’= 0.57 ± 0.11), in Chilean (H’ = 0.64 ± 0.18) than in the foreign (H’ = 0.55 ± 0.12) germplasm, and in modern (H’ = 0.62 ± 0.15) than in historic (H’ = 0.55 ± 0.12) germplasm (**Figure 5A**). As expected, phenotypic diversity was lower in the food-oat breeding germplasm (H’ = 0.42 ± 0.15) than in the pre-breeding (H’ = 0.65 ± 0.19) group.

The phenotypic performance of the germplasm for food-oat breeding was better in Cluster 2 (MGIDI = 5.34 ± 0.91) and Cluster 3 (MGIDI = 5.63 ± 1.00), than for Cluster 1 (MGIDI = 7.91 ± 1.13); there was a tendency to a better performance in Cluster 2 than in Cluster 3 (*P* = 0.071) (**Figure 5B**). Also, the phenotypic performance was superior in Pop 2 (MGIDI = 5.19 ± 0.95) than Pop 1 (MGIDI = 5.97 ± 1.19), in Chilean (MGIDI = 5.34 ± 1.08) than in foreign (MGIDI = 6.06 ± 1.14) germplasm (*P* < 0.001), and in modern (MGIDI = 5.34 ± 6.36) than in historic (MGIDI = 6.36 ± 1.12) germplasm (*P* < 0.001). As expected, phenotypic performance was higher in the food-oat breeding (MGIDI = 4.23 ± 0.39) than in pre-breeding (MGIDI = 6.03 ± 1.00) selected germplasm (*P* < 0.001).

The genetic diversity was similar (*P* > 0.05) in the three phenotypic clusters, between the Chilean (He = 0.53 ± 0.11) and foreign (He = 0.59 ± 0.16) germplasm, and between historic (He = 0.54 ± 0.14) and modern (He = 0.59 ± 0.13) germplasm (**Figure 5C**). As expected, genetic diversity was higher (*P* = 0.035) in Pop 1 (He = 0.59 ± 0.04) than Pop 2 (He = 0.44 ± 0.05). A tendency to lower genetic diversity (*P* = 0.09) was observed in the germplasm selected for food-oat breeding (He = 0.48 ± 0.18) compared to the pre-breeding (He = 0.59 ± 0.13) germplasm. Different number of private alleles were found; they were present in Cluster 1 (N = 3) and Cluster 2 (N = 1), Pop 1 (N = 14), foreign (N = 6) and Chilean (N = 2) germplasm, in pre-breeding (N = 17) selected germplasm and in historic (N = 6) genotypes (**Supplementary Table S2**). However, private alleles were absent in Pop 2, Cluster 3, modern germplasm and in food-oat breeding selected germplasm (**Supplementary Table S2**).

**Figure 6:**
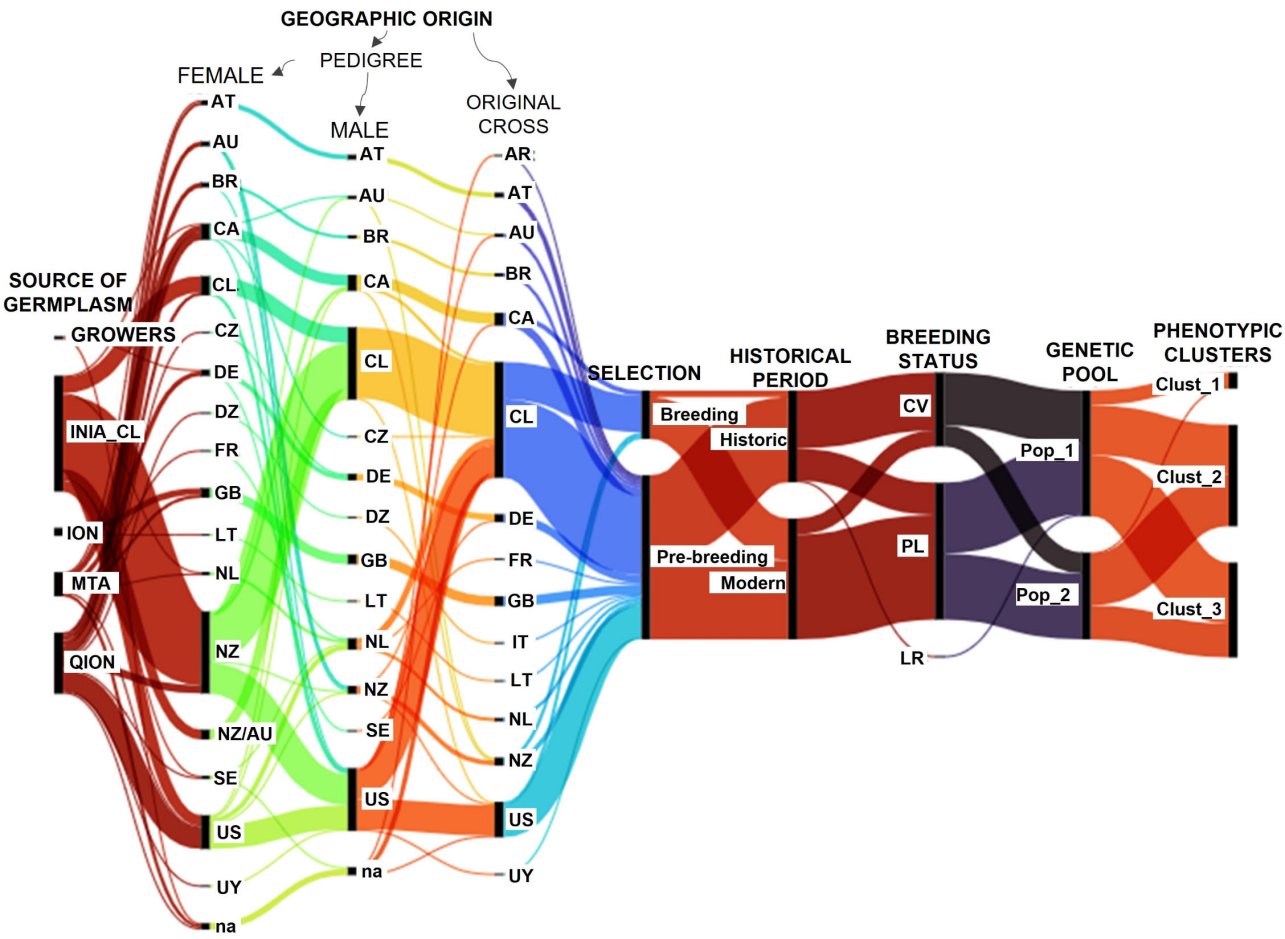
Relationships of the oat genotypes according to their geographic origins and grouping patterns. QION, Quaker International Oat Nursery; ION, International Oat Nursery; MTA, material transfer agreement. CV, cultivar; PL, pure line; LR, land race. R, Argentina; AT, Austria; AU, Australia; BR, Brazil; CA, Canada; CL, Chile; CZ, Czech Republic; DE, Germany; DZ, Africa; FR, France; GB, United Kingdom; IT, Italy; LT, Lithuania; na, not assigned; NL, Netherlands; NZ, New Zealand; SE, Sweden; US, United States of America and UY, Uruguay. The Chilean germplasm corresponds to genotypes created in Chile using diverse origin germplasm. Historic: germplasm created on or before 2010, and modern: after 2010.

The alluvial diagram allowed us to visualize the origins of the oat genotypes and group them according to their phenotypic similarity and genetic pools (**Figure 6**). The germplasm showed a complex interrelated pattern, reflecting a germplasm mostly created from parents with diverse origins. The germplasm was formed mainly by Chilean accessions and others from the USA and Canada, and a low proportion from other countries of South America (Argentina, Uruguay, Brazil), Europe (Germany, France, United Kingdom, Italy, Lithuania, Netherland, Austria), and Oceania (New Zealand, Australia), representing 15 different geographic origins.

Thirty-six out of 46 cultivars, 20 out of 86 pure lines and the Rubia Corriente landrace are historic genotypes created 2010 or earlier (**Figure 6**). Cultivars were grouped in higher proportion in Pop 1 (N = 33) than Pop 2 (N = 13), while pure lines were in similar proportions in Pop 1 (N = 44) and Pop 2 (N = 41), resulting in a higher proportion of historic germplasm in Pop_1 (59%) than Pop_2 (20%). Pop_1 contained nine out of 10 genotypes from Cluster 1, and a slightly lower number of genotypes from Cluster 2 (N = 31) than Cluster 3 (N = 38), while Pop 2 contained only one genotype from Cluster 1 and more genotypes from Cluster 2 (N = 32) than Cluster 3 (N = 21), explaining the better phenotypic performance of Pop 2 in relation to Pop 1. Consequently, the germplasm selected for food-oat breeding corresponded mainly to modern germplasm belonging to Pop 2 and Cluster 2.

## 4 Discussion

Oats have gained popularity worldwide since oat-based products consumption can contribute to lower cholesterol and diabetic effects, preventing disease and promoting human health (Paudel et al., 2021). This cereal ranks second in cultivated area in Chile; it is mainly an export product (ODEPA, 2023), and as such is economically important for local oat producers and processing industries. During 2010-2023 period, 70 to 90% of the Chilean oat cropping area has been covered with cv. Supernova INIA (De la Fuente, 2022). The wide spread of dominant cultivars, generating continuous low crop genetic diversity and high uniformity, could lead to crop vulnerability (Khoury et al., 2022; Salgotra and Chauhan, 2023), thus the integration of new genetic diversity associated with favorable commercial traits is essential to increase resilience against climate change (Swarup et al., 2021), and to deal with the emergence of new biotic and abiotic stresses (Ristaino et al., 2021; Skendžić et al., 2021; FAO, 2022). A way to increase diversity in modern cereal cultivars, highly uniform and sown in large areas like oats, is the quick turnover of cultivars (Khoury et al., 2022). However, none of the cultivars released in Chile and new advanced lines created have significantly outperformed Supernova INIA, resulting in low adoption by producers and a low release rate of new cultivars. Therefore, quantifying and broadening the available genetic diversity are expected to be critical to increase the rate of cultivar turnover in Chile.

A study of genetic diversity of the oat germplasm available in INIA, based on molecular markers and including new advanced lines and commercial cultivars was missing, because the selection was mostly based on agronomic performance. A comparable phenotypic evaluation of the germplasm, including historic and modern genotypes of diverse origin, was also missing due to disconnected evaluations in different historical periods. For these reasons, we analyzed the phenotypic and genetic diversity of 132 oats accessions with 28 traits and 14 SSR markers, in a field trial sown in La Araucanía Region, where the main oat cropping area is concentrated. Important genetic parameters affecting the success of genetic breeding were also estimated. The results obtained here supported the applicability of most of the current food-oat breeding objectives, and consequently promise good prospects for obtaining new cultivars in the short term. However, some noteworthy issues were identified which could make breeding from the currently available germplasm difficult in the long term.

Moderate genetic diversity and a discrete population structure were found in the studied germplasm, similar to most foreign germplasm analyzed worldwide (Nersting et al., 2006; Jan et al., 2020; Lyubimova et al., 2020; Wang et al., 2023), showing a low rate of diversification of the germplasm. The Chilean germplasm showed similar genetic diversity in comparison to foreign germplasm from 14 different origins. The phenotypic diversity of the germplasm in key agronomic and grain quality traits accounted for a low proportion of genotypes with favorable phenotype in relation to the reference cultivar Supernova INIA, showing a high grade of fixation of these traits under intensive selection in the breeding program. These factors could explain the relatively low success of the breeding efforts taking place in Chile. Despite these negative aspects, a group of 26 superior genotypes compared to Supernova INIA, mostly modern Chilean pure lines, were selected by a multi-trait index calibrated for commercial food-oat breeding. These genotypes could play an important role in widening the genetic diversity of the oat crop in Chile if some of them pass the necessary tests for release as new cultivars.

The rest of the genotypes of lower performance for food-oat breeding compared to Supernova INIA are a valuable genetic resource for pre-breeding and further studies on other characters not addressed in the present study. Thus, the conservation of this germplasm is relevant. These results emphasize the key role of using diverse origin germplasm and carrying out genetic breeding in Chile, since almost all the selected superior genotypes were created with foreign germplasm but selected in the southern Chile environment. It has been proposed that investment in public breeding programs, providing pre-breeding and other diversification services to formal seed systems, also of advanced breeding technologies (e.g. genetic modification and gene editing), will be needed to mitigate negative impacts on modern cultivar diversity (Khoury et al., 2022).

### 4.1 Phenotypic variation and heritability of the traits

Phenotypic variation is the distribution or range of morphological, phenological, developmental and biochemical traits that are expressed in individual taxa (Kalisz and Kramer, 2008), determining the capacity of plants to adapt to changing environments and to colonize new habitats (Boquete et al., 2021). Having populations with a high range of phenotypic variability is indispensable to achieve genetic gain in breeding programs (Swarup et al., 2021). For this reason, we evaluated the phenotypic variation of the germplasm in terms of dispersion of the data (genetic coefficient of variation, CVg) and range of variation. Low CVg (< 6%) was found in hectoliter weight, groat content and heading days, while the 25 other traits exhibited moderate to high CVg, in most cases in an adequate range of variation for a selection program. The range of variation in phenotypic traits can broadly vary depending on the germplasm and environmental conditions. For example, the ranges of variation observed in heading days (79.33-97 d, 41-69 d), thousand hulled grain weight (6-27.33 g, 19-45.7 g) and plant height (52-134.7 cm) in 38 oat Indian genotypes (Kumar et al., 2023), and in 64 European oat cultivars and 17 landraces (Nersting et al., 2006), respectively, were of lower magnitude in comparison to the present study.

Heritability is a key concept in breeding, corresponding to the fraction of the total variance among plants of a population that may be attributed to genetic differences between them, which is one of the most important components of the breeder’s equation that aims to predict the expected response to selection (Mazurkievicz et al., 2019). Most traits studied here exhibited moderate to high heritability (H^2^ > 0.50), indicating a good prospect to achieve genetic gain in a selection program. The larger the heritability of a trait, the greater the expected genetic gain, in which case artificial selection can be carried out more efficiently (Mazurkievicz et al., 2019). The low heritability (H^2^< 0.40) observed for grain yield, plant height, vigor scores at tillering, the incidence of diseases like *P. syringae*, Barley Yellow Dwarf Virus, and hull staining, reflects the large influence of environmental factors affecting these traits. Selection may be difficult in traits with low heritability (< 0.40), due to the confounding effect of the environment (Majhi, 2020).

Several studies on heritability of oat traits have been published, with heading days, plant height at maturity, and thousand hulled grain weight, being the most commonly traits investigated (Sürek and Valentine, 1996; Yan et al., 2016; Kumari et al., 2017; Premkumar et al., 2017; Chauhan and Singh, 2019; Leišová-Svobodová et al., 2019; Mazurkievicz et al., 2019; Meira et al., 2019; Haikka et al., 2020; Vanjare et al., 2021; Brzozowski et al., 2022). However, heritability cannot be generalized to a crop since it is highly specific, and valid only for the material involved in the experiment and the experimental environment (Majhi, 2020). High variation in the results has been observed in oats, depending on the type of germplasm, management, and experimental design of the trials (**Supplementary Table S9**). The heritability in other studies included 12 of the 28 traits studied here, including heading days (0.46-0.94), plant height at maturity (0.50-0.98), panicle length (0.12-0.86), thousand hulled (0.26-0.97) and thousand dehulled (0.30-0.94) grain weights, grain yield (0.00-0.88), groat protein (0.88-0.93), groat fat (0.13-0.99), groat content (0.23-0.91), hectolitre weight (0.89), panicle type (0.68) and hull color (0.88); the values obtained here were mostly within the ranges observed in aforementioned studies (**Supplementary Table S9**). We are contributing with broad sense heritability data for 16 oat traits not reported in the reviewed literature.

### 4.2 Phenotypic diversity

We used the scaled Shannon-Weaver diversity index (H’) to quantify the phenotypic diversity of the germplasm on mostly quantitative traits, except for panicle type and hull color, which are qualitative traits. A low H’ indicates extremely unbalanced frequency classes for an individual trait and a lack of diversity (Upadhyaya et al., 2002). Almost all the traits exhibited high phenotypic diversity (H’ > 0.6), with exception of hectoliter weight and groat dry matter (H’ < 0.3). Hectoliter weight, groat content and thousand hulled grain weight, which are important quality traits for the industry, are mostly fixed in the germplasm in relation to the reference cultivar Supernova INIA. Consequently, these results show possibilities of maintaining but not substantially increasing the quality of future cultivars.

There was high diversity in heading days (H’ > 0.7) but the germplasm was mainly in the range of midseason to long cycles, which might make it hard create earlier cultivars. Grain yield and plant height at maturity showed a high diversity; about 15% of the germplasm had higher yield and shorter plant height compared to Supernova INIA. However, since grain yield exhibited low heritability, low genetic gain is expected. The disease scores and new implemented grain quality traits such as high severity and low severity groat staining, broken grains after peeling and groat protein and fat contents had high diversity, with a good proportion of genotypes with favorable phenotype *versus* Supernova INIA, thus there are good prospects for improvement of these traits in new cultivars.

There are few studies in oats reporting diversity indexes. For instance, diversity (H) was lower (0.9-1.4) in a group of 84 Nordic oat cultivars and landraces, considering three traits (Nersting et al., 2006). In a group of 91 Polish oat landraces which included eight traits, H was only slightly lower (0.79-2.08) (Boczkowska et al., 2016) than in this study (0-2.55). The studied oat germplasm exhibited high overall phenotypic diversity (H’ > 0.6) (Yemataw et al., 2018), considering a wider number of phenotypic traits and oat genotypes compared to the available literature.

### 4.3 Pairwise correlations between traits

Quantitative traits often exhibit complex inter-relationships which can hinder breeding for multiple traits at a time. For this reason, understanding the associations between traits is a prerequisite to approach breeding (Yan et al., 2016). Non-zero genetic correlations can occur by close linkage of loci, or by pleiotropy which occurs when two traits are controlled by the same loci (Bernardo, 2020). Unlike linkage-correlations, pleiotropic-correlations cannot be dissipated by repeated cycles of meiosis, due to their physiological bases (Bernardo, 2020). The ability to differentiate between pleiotropy and close-linkage correlations will determine the optimum breeding strategy (Chen and Lübberstedt, 2010).

The nature of the correlations in oats is still poorly understood, shown by the scarcity of related literature. Thus, with the purpose of generating validated information on diverse origin oats, the pairwise Pearson correlations (r) between all the traits routinely measured in the oat breeding program were calculated, resulting in 108 weak to moderate significant associations (*P* ≤ 0.05). However, a significant value of r does not imply causality or a direct relationship between the variables, since two variables can be correlated because both have a significant degree of correlation with a third variable (Armstrong, 2019). To avoid this confusion, partial correlation coefficients were computed to corroborate the results. Partial correlations have been useful when multiple variables are present because they consider the effect of a third confounding variable on the correlation (Armstrong, 2019; Olivoto and Lúcio, 2020). As expected, only 44 significant associations were confirmed by the partial correlation analysis, the remaining 64 associations were caused by other confounding variables. Interestingly, 18 significant partial correlations were not detected by pairwise correlations, showing the ability of the partial analysis to detect associations hidden because of other confounding variables.

The trait correlations found in most studies used fewer traits and different comparisons (Khan et al., 2014; Crestani et al., 2015; Boczkowska et al., 2016; Baye et al., 2020), making comparison to our results difficult. The significant associations we found with pairwise and partial correlations were mostly favorable for breeding purposes, indicating good possibilities to improve these traits simultaneously, with exception of groat protein content, which showed unfavorable associations with key traits like grain yield. Similar results for the negative association between grain yield and groat protein (r = -0.38, *P* < 0.01) and opposite result for the negative association between protein and groat content (r = 0.13, *P* < 0.05), were observed in two biparental oat populations in different environments and seasons; there were descendants with appropriate combinations of these traits and transgressive segregation (Yan et al., 2016). Since the unfavorable associations here were of small biological importance, they should not be a limitation for genetic improvement if good combinations of traits are identified in the germplasm, together with a continued effort to develop the desired cultivars (Yan et al., 2016).

### 4.4 Phenotypic clustering of the oat germplasm

A principal component analysis showed the distribution of the genotypes (distances in the factor map) and the combination of the traits in the oat germplasm (correlations). The high phenotypic diversity of the germplasm was evident. A cluster analysis of the principal coordinates grouped the genotypes exhibiting similar phenotypic patterns into three clusters. Depending on whether the trait mean in the cluster was higher or lower compared to the overall mean of the total population, the trait was considered favorable or unfavorable for food-oat breeding. Cluster 1 grouped mainly oats with aptitude for feed, markedly different than the rest of genotypes due their very high plant heigh and straw weakness, such as the landrace Rubia Corriente widely used in Chile as fodder, silage, and grain in animal production (Beratto, 1977), among others.

The remaining germplasm was separated in similar proportion in Cluster 2 and Cluster 3. Cluster 2 had high values of grain yield, agronomic scores, industrial grain quality and longer open panicles, whereas Cluster 3 grouped genotypes with low plant height and low lodging, high protein content, low industrial grain quality, and short compact panicles. These two phenotypic patterns show limitations to breed dwarf and lodging-tolerant varieties combined with high yield and high industrial grain quality, since these phenotypes were not observed in the germplasm, independently of the breeding status (pure lines, cultivars), geographic origins (Chilean, foreign), and historical period (historic, modern). Creating a fourth group, combining positive characteristics of Cluster 2 and Cluster 3, would allow diversifying the phenotypes of the available oat germplasm.

### 4.5 Phenotypic value of the oat germplasm for food-oat breeding

We estimated the BLUP phenotypic values of the germplasm, corresponding to the best linear neutral prediction for estimating genetic competence (Mahdi and Mohammad, 2022), accounting for both the additive and nonadditive genetic effects of a line (Henderson, 1976). This is critical information for parental selection decisions and for determining the relative “eliteness” of a line (Cobb et al., 2019). The simultaneous selection for multiple traits and different breeding objectives made it hard to visualize the eliteness of the oat genotypes. For this reason, we used the MGIDI, a BLUP-based selection index which carried the correlations between traits to a single plane, considering the breeding objectives (increase-decrease), and the intra-mean traits heritability in the estimation of genetic gain (Olivoto and Nardino, 2021).

Only 26 genotypes, 20% of the total germplasm, outperformed the elite phenotype of the reference cultivar Supernova INIA for food-oat breeding. These genotypes would be valuable in the diversification of oat crops in Chile if some of them are released as cultivars and/or used in crosses. However, the lower diversity of the selected genotypes compared to the rest of the germplasm, and the qualities for breeding in the selected group in relation to Supernova INIA, confirmed the convenience of a more diverse germplasm in favorable traits than that currently available in the breeding program at INIA-Chile.

The germplasm with lower performance than Supernova INIA was discarded for commercial breeding but selected for pre-breeding, because these genotypes exhibited specific traits useful for development of new cultivars. The introduction of genetic diversity from genotypes that have contrasting phenotypic traits is a major challenge; there are numerous examples in plants showing an unfavorable phenotype due to adverse genetic background effects and linkage drag with the desired trait (Swarup et al., 2021). Since the INIA breeding program is mainly based on bi-parental crosses, the descendants will contain up to 50% of the genome of each parent, unfavorable phenotypes being expected using parents of low phenotypic performance. Consequently, a long-term pre-breeding process will be required to reduce the negative effects in new cultivars.

### 4.6 Population structure

The clustering of the oat germplasm based on genetic distance estimated with 14 SSR markers separated the germplasm into two genetic subtrees, although with low bootstrap support. A Bayesian approach that estimates for each accession the proportion of the genome that originates from each subpopulation, also called percentage of admixture (Pritchard et al., 2000; Montilla-Bascón et al., 2013), together with Evanno´s statistic (Evanno et al., 2005), detected the existence of two different subpopulations or genetic pools, called Pop 1 and Pop 2; Pop 1 included Rubia Corriente, Llaofén INIA, Eva and Júpiter INIA, while Pop 2 was the genetic pool of Supernova INIA and Urano INIA. Interestingly, Pop 1 grouped most historical and foreign germplasm, whereas Pop 2 was mostly composed of modern Chilean pure lines, commercial cultivars available in Chile and a few foreign cultivars, suggesting that allele combinations in Pop 2 would be associated with better agronomic performance in the southern Chile environment.

The oat germplasm exhibited a weak population structure, with an optimal number of two sub-populations. A Structure analysis on a collection of 141 *Avena sativa* L. landraces including 110 white, and 31 red oats from Spain based on 31 SSRs supported the existence of two gene pools (Montilla-Bascón et al., 2013). The same happened with 24 landraces from India studied with 24 SSRs (Rana et al., 2019); in 85 oats with white, yellow, and brown seeds as well as a subgroup of naked oats from 18 different countries based on seven SSRs (Havrlentová et al., 2021); in 91 indigenous accessions from Poland using eight ISSR markers (Boczkowska et al., 2016); on 288 oats genotypes of diverse origin using 2143 SNPs (Wang et al., 2023); in 487 *Avena sativa* accessions mainly from Poland using 7411 SNPs (Koroluk et al., 2023); and in 38 oat accessions from India using 22 ISSRs (Kumar et al., 2023). Structure supported three subpopulations in a group of 1,000 world-wide oat accessions, including cultivars, germplasm of uncertain improvement status, and landraces, based on data from 2,715 SNP markers (Winkler et al., 2016), and in a 260 diverse origin collection of husked, naked and black oats using 15 SSRs (Leišová-Svobodová et al., 2019). Thus, in different oat germplasms and applying different types and number of markers, the population structure was essentially formed by two genetic pools. The low level of differentiation between populations shows a low level of diversification of the oat germplasm. This might be due to the recent domestication of *Avena sativa* L., which appeared in cultivation several thousand years later than wheat and barley (Zohary and Hopf, 2000).

### 4.7 Genetic variation and diversity

Genetic variation is related to differences in particular DNA sequences between individuals, while genetic diversity is related to DNA differences in populations (Swarup et al., 2021). It is said that permanent access to genetic variation for different phenotypic traits is a requisite for obtaining long-term breeding progress (Swarup et al., 2021), whereas genetic diversity is the main driving force for the selection and evolution of populations (Salgotra and Chauhan, 2023). The oat genotypes were well differentiated, showing variability based on their genetic distances except for two non-differentiable duplicates. The average PIC (0.58), average number of alleles per locus (4.3) and number of rare alleles per locus (1.42), were lower than in other studies, such as 177 white and red oats land races and cultivars from Spain characterized with 31 SSRs, which had an average PIC of 0.80, 14.45 alleles and 4.45 rare alleles per locus (Montilla-Bascón et al., 2013). The average genetic diversity of the germplasm was moderate (He = 0.52 ± 0.03); it was 15% to 28% higher in Pop 1 than in Pop 2, depending on the index. Pop_1 retained 100% of the allele richness *versus* Pop 2 accounting for 75.4%; private alleles were only found in Pop 1. Thus Pop 1 is a reservoir of genetic diversity for oat pre-breeding.

The genetic diversity of this oat germplasm was difficult to compare with other studies due to the different molecular markers used, population sizes and origins. Having said that, similar genetic diversity (He) compared to the present study was obtained in a group of 16 exotic oat genotypes from Europe and Pakistan (0.12-0.53) using five RAPD primers with 23 loci amplified (Jan et al., 2020); in 18 oat cultivars from Russia, Norway, Netherlands, and Sweden (0.33-0.75) based on avenin-like alleles (Lyubimova et al., 2020); in 64 oat cultivars from Europe (0.5-0.63) using seven SSRs (Nersting et al., 2006); in 288 oat genotypes from diverse origin (0.096-0.50) using 2143 SNPs (Wang et al., 2023); and in a 260 naked, husked and black oats collection (0.48-0.61) using 15 SSRs (Leišová-Svobodová et al., 2019). However, low genetic diversity was found in 23 oat cultivars from Poland (0.20) using the dominant markers ISSR, RAPDs and AFLPs (Boczkowska et al., 2016), in 177 red and white oats from southern Spain (0.29) using 31 SSRs (Montilla-Bascón et al., 2013), in 72 Polish oats (0.15-0.30) using 36 ISSRs (Koroluk et al., 2022), and in 60 accessions representing 13 *Avena* species (0.000-0.068) using retrotransposon primer binding sites-iPBS (Androsiuk et al., 2023). Thus, the mostly moderate genetic diversity observed here, which is coincident with most other studies from around the world, shows a limitation for oat breeding, indicating the need to increase the diversity using different strategies to ensure genetic gain in the long term.

Most studies of genetic diversity in oats, including the present work, used binary data to estimate genetic parameters and population structure, applying different types of markers (SSRs, ISSR, RAPD, AFLP, iPBS), masking the size and sequence of alleles. In the case of SSRs, the length of the alleles has served to approximate the number of repetition units, and has been used to calculate genetic and evolutionary distance between individuals (Šarhanová et al., 2018). Additionally, SSRs fragments of the same size but with different sequences, frequently referred to as size homoplasy, have been revealed through sequencing (Estoup et al., 2002). Thus, the use of binary data could cause alleles misinterpretation and bias in genetic parameters. For example, in 1135 samples of different populations of *Ceratonia siliqua* (Leguminosae), sequence-based SSRs genotyping allowed for a better estimate of population divergence, detecting higher private allele richness compared to size fragments scoring (Viruel et al., 2018). In another study, SSRs fragment sequences revealed higher number of alleles and higher genetic diversity, but a similar F_ST_, in comparison to fragment size scoring in 384 accessions of *Donatia fascicularis* (Stylidiaceae), 88 accessions of *Mulguraea tridens* (Verbenaceae), and 384 accessions of *Oreobolus obtusangulus* (Cyperaceae) (Šarhanová et al., 2018). On the other hand, a thorough review of homoplasy in diverse molecular ecology studies, concluded that homoplasy does not represent a significant problem in most types of analyses of population genetics, because it is often compensated by a large amount of variability in microsatellite loci (Estoup et al., 2002). Therefore, whilst including information of alleles sequences or size might have allowed detecting greater variation and improving the accurateness of populations structure and genetic diversity estimates, we consider the current results as reliable. For the purposes of the present work, the SSRs markers coded as binary data were a cost-effective way to estimate genetic parameters in oats, accepting that a rate of unknown bias might exist. We are not aware of any published studies quantifying homoplasy in oats. Comparing different markers platforms, SSRs still represent a useful marker system because of their high mutation rates and cost-effectiveness (Viruel et al., 2018), being recommended for projects with limited budgets (Jennings et al., 2011), such as the present one.

### 4.8 Perspectives for oat breeding in Chile

Oat breeding in Chile has had slow progress after the release of cv. Supernova INIA, although the breeding program has been permanently introducing new germplasm of diverse origin. The similar genetic diversity, and higher phenotypic diversity and value of the Chilean germplasm, compared to the available foreign germplasm studied here, could explain the relatively poor success. Similar diversity status and higher phenotypic performance was observed in modern germplasm compared to historical genotypes. This reflects the efforts made in the last decade by the INIA breeding program aiming to release new lines with a better performance in comparison to imported and historic cultivars. Maintaining genetic diversity in a breeding program is essential to guarantee sustainable genetic gain for targeted traits (Salgotra and Chauhan, 2023; Sanchez et al., 2023). Both the Chilean and modern germplasm studied here conserved similar genetic diversity in comparison to the foreign and historical germplasm, harboring a similar magnitude of diversity in relation to other oat germplasms evaluated in other studies. Thus, a good prospect for genetic improvement is seen in the short term if the use of these genetic resources is optimized. The introduction of new sources of diversity is relevant in this regard, to avoid reaching a genetic gain plateau in the longer term (Sanchez et al., 2023).

The germplasm studied here exhibited two genetic subpopulations with a weak differentiation, showing a low genetic divergence in the germplasm, and reflecting the short domestication period of oats (Zohary and Hopf, 2000). Genetic divergence can be due to mutation, genetic drift, and selection (Kozak et al., 2011), and is important in plant breeding to identify and utilize genetic variation within and between populations to develop new and improved varieties (Dhanalakshmi et al., 2023). Pop 1 was identified as a reservoir of allele richness and genetic diversity, whereas Pop 2 showed slightly lower genetic diversity, probably caused by elimination of inferior alleles during selection, deduced from their superior overall phenotypic performance compared to Pop 1, and the lack of private alleles in Pop 2.

If the divergence between subpopulations is neutral regarding the frequency of favorable alleles over multiple loci, the best hybrids are more likely to come from inter-population crosses (Mackay et al., 2021). Despite the higher phenotypic performance of Pop 2, examples of oats with good phenotypic value were also found in Pop 1, which would be good parental candidates for implementing between-population crosses, and at the same time achieve a higher dispersion of favorable alleles. The dispersion of favorable alleles between parents causes transgressive segregation, in which progenies exhibit a phenotype outside the range of the parents (Mackay et al., 2021). Importantly, phenotypes produced by transgressive segregation are heritably stable and can be observed in crosses involving parents of proximal phenotypes, as for example heading days in rice, due to the existence of hidden genetic variation between the parents (Koide et al., 2019). Several foreign pure lines included in this study exhibited acceptable phenotypic values and belonged to different genetic subpopulations. These lines have not yet been used in crosses, or in other cases, their progenies are still in preliminary evaluation stages in Chile. The selection of transgressive genotypes in crosses implemented with the information generated in this study, should be the next step for generating new improved lines in the short term with the available germplasm. It is projected that this strategy would be useful to express transgressivenness in important characters for food-oat breeding, exhibiting a similar but not superior phenotype compared to supernova INIA in the studied germplasm, such as hectoliter weight, heading days, groat content, and plant height, among others.

A two-way strategy is proposed for long term breeding, based on the observed results. First, the enrichment of the genetic diversity to overcome the diversity plateau can be approached through a continuous exchange of germplasm, which would be dependent on the accessibility of new genetic resources. This path can also be explored through the creation of new genetic diversity through strategies such as genetic modification, and gene-editing and mutation-breeding. Transformation of oats using biolistic bombardment improved the tolerance to osmotic stress in transgenic plants (Maqbool et al., 2009). Gene-editing has been successfully applied in the breeding of wheat, rice, and barley to improve tolerance to biotic and abiotic stresses, grain quality and yield (Riaz et al., 2022). However, despite the usefulness of these modern techniques in genetic breeding, they are not currently accepted for food production by regulatory agencies in Chile. Alternatively, mutation is a non-transgenic powerful approach to generate novel genetic variation that can be exploited by breeding programs (Abaza et al., 2020). At least two thousand rice, barley, wheat, soybean, maize, and oat mutant varieties have been released to farmers (https://nucleus.iaea.org/sites/mvd/SitePages/Home.aspx). The development of Targeting Induced Local Lesion IN Genomes (TILLING), consisting in mutagenesis followed by a rapid identification of mutations in the genes of interest (Szurman-Zubrzycka et al., 2023), has been broadly used to improve traits such as starch synthesis, plant architecture, disease resistance, drought and salinity tolerance, and other yield parameters in cereals (Irshad et al., 2020; Nouman-Khalid et al., 2021; Abdelnour-Esquivel et al., 2010). In oats, TILLING allowed to induce variation in genes encoding for enzymes involved in the pathways of lignin and beta-glucan biosynthesis (Chawade et al., 2010). Recently, mutagenesis by direct current electrophoresis bath (DCEB) was investigated in rice (Zou et al., 2023). Mutation by exposing seeds or plants to cosmic radiation in outer space, or space-breeding, has allowed the release of at least 66 varieties in China (Liu et al., 2009; Mohanta et al., 2021). Second, the breeding program can be optimized through continuous monitoring of existing genetic diversity, improving selection of parents in crosses to maintain a high diversity level. It is well known that investment in oat breeding programs on a global scale has been rather low, as shown by fewer published articles related to new breeding technologies in the species compared to other cereal crops like wheat and barley. This fact might limit the implementation of modern breeding tools in oats also in Chile.

## 5 Conclusion

The oat germplasm studied here contained high phenotypic diversity but with a discrete proportion of genotypes exhibiting adequate phenotypic performance for food-oat breeding compared to Supernova INIA, the most cropped cultivar in Chile. This, together with the higher phenotypic performance and similar genetic diversity of the Chilean germplasm compared to foreign germplasm, explain in part the slow progress of Chilean breeding after Supernova INIA, even with continuous introduction of new germplasm. Heritability, range of variation and correlations of phenotypic traits in the studied germplasm shows an auspicious genetic breeding, for most food-oat breeding objectives. However, the germplasm studied here showed moderate genetic diversity, with two weakly differenced genetic pools, similar to other oat germplasms studied around the world, reflecting the low genetic divergence in the species. These factors underline the urgent need to enrich the genetic and phenotypic diversity of the currently available germplasm, making efficient use of the genetic resources, integrating the results obtained here in making decisions to maintain high diversity in the breeding program. In the long term, investing in modern breeding tools or mutation breeding to overcome the diversity plateau in the species, is proposed as an alternative independent of the availability of foreign genetic resources to enrich the species diversity in breeding programs. The results of the present study depict a challenging prospective for oat breeding in Chile.

## Data availability statement

The raw data supporting the conclusions of this article will be made available by the authors, without undue reservation.

## Author contributions

MM: Formal analysis, Funding acquisition, Project administration, Supervision, Writing – original draft, Conceptualization, Data curation, Investigation. VP: Investigation, Writing – review & editing. MM: Writing – review & editing. FF: Investigation, Writing – review & editing. AV: Investigation, Writing – review & editing. IL: Investigation, Methodology, Writing – review & editing. MS: Methodology, Investigation, Writing – review & editing. RS: Investigation, Writing – review & editing. PH: Investigation, Supervision, Writing – review & editing.

## References

[B1] Abaza G. Awaad H. A. Attia Z. M. Abdel-lateif K. S. Gomaa M. A. Safy M.. (2020). Inducing potential mutants in bread wheat using different doses of certain physical and chemical mutagens. Plant Breed. Biotech. 8, 252–264. doi: 10.9787/PBB.2020.8.3.252

[B2] Abdelnour-Esquivel A. Perez J. Rojas M. Vargas W. Gatica-Arias A. (2010). Use of gamma radiation to induce mutations in rice (*Oryza sativa* L.) and the selection of lines with tolerance to salinity and drought. In Vitro Cell.Dev.Biol.-Plant 56, 88–97. doi: 10.1007/s11627-019-10015-5

[B3] Achleitner A. Tinker N. A. Zechner E. Buerstmayr H. (2008). Genetic diversity among oat varieties of worldwide origin and associations of AFLP markers with quantitative traits. Theor. Appl. Genet. 117, 1041–1053. doi: 10.1007/s00122-008-0843-y 18633590

[B4] Amiryousefi A. Hyvönen J. Poczai P. (2018). iMEC: online marker efficiency calculator. Appl. Plant Sci. 6, e01159. doi: 10.1002/aps3.1159 30131901 PMC6025818

[B5] Androsiuk P. Milarska S. E. Dulska J. Kellmann−Sopyła W. Szablińska−Piernik J. Lahuta L. B. (2023). The comparison of polymorphism among *Avena* species revealed by retrotransposon−based DNA markers and soluble carbohydrates in seeds. J. Appl. Genet. 64 (2), 247–264. doi: 10.1111/opo.12636 36719514 PMC10076396

[B6] Armstrong R. A. (2019). Should pearson's correlation coefficient be avoided? Ophthalmic Physiol. Opt. 39, 316–327. doi: 10.1111/opo.12636 31423624

[B7] Arora A. Sood V. Chaudhary H. Banyal D. Kumar S. Devi R.. (2021). Genetic diversity analysis of oat (*Avena sativa* L.) germplasm revealed by agro-morphological and SSR markers. Range Manage. Agrofor. 42, 38–48.

[B8] Baye A. Berihun B. Bantayehu M. Derebe B. (2020). Genotypic and phenotypic correlation and path coefficient analysis for yield and yield-related traits in advanced bread wheat (*Triticum aestivum* L.) lines. Cogent Food Agric. 6, 1752603. doi: 10.1080/23311932.2020.1752603

[B9] Beratto E. (1977). Efectividad de la selección por línea pura en el mejoramiento de avena Rubia corriente. Agric. Téc. 37, 150–155. Available at: https://hdl.handle.net/20.500.14001/36140 (Accessed December 12, 2023).

[B10] Beratto E. (2006). Cultivo de la avena en Chile (Temuco, Chile: Colección Libros INIA - Instituto de Investigaciones Agropecuarias. no. 19).

[B11] Bernardo R. N. (2020). Breeding for quantitative traits in plants. 3rd ed. (Woodbury, Minnesota: Stemma Press).

[B12] Boczkowska M. Łapiński B. Kordulasińska I. Dostatny D. F. Czembor J. H. (2016). Promoting the use of common oat genetic resources through diversity analysis and core collection construction. PLoS One 11, e0167855. doi: 10.1371/journal.pone.0167855 27959891 PMC5154523

[B13] Boquete M. T. Muyle A. Alonso C. (2021). Plant epigenetics: phenotypic and functional diversity beyond the DNA sequence. Am. J. Bot. 108, 553–558. doi: 10.1002/ajb2.1645 33887061

[B14] Brzozowski L. J. Hu H. Campbell M. T. Broeckling C. D. Caffe M. Gutiérrez L.. (2022). Selection for seed size has uneven effects on specialized metabolite abundance in oat (*Avena sativa* L.). G3 Genes Genomes Genet. 12, jkab419. doi: 10.1093/g3journal/jkab419 PMC9210299 34893823

[B15] Chauhan C. Singh S. (2019). Genetic variability, heritability and genetic advance studies in oat (*Avena sativa* L.). Int. J. Chem. Stud. 7, 992–994.

[B16] Chawade A. Sikora P. Bräutigam M. Larsson M. Vivekanand V. Nakash M. A.. (2010). Development and characterization of an oat TILLING-population and identification of mutations in lignin and beta-glucan biosynthesis genes. BMC Plant Biol. 10, 86. doi: 10.1186/1471-2229-10-86 20459868 PMC3017761

[B17] Chen Y. Lübberstedt T. (2010). Molecular basis of trait correlations. Trends Plant Sci. 15 (8), 454–461. doi: 10.1016/j.tplants.2010.05.004 20542719

[B18] Cieplak M. Okoń S. Werwińska K. (2021). Genetic similarity of *Avena sativa* L. varieties as an example of a narrow genetic pool of contemporary cereal species. Plants 10, 1424. doi: 10.3390/plants10071424 34371627 PMC8309341

[B19] Cobb J. N. Juma R. U. Biswas P. S. Arbelaez J. D. Rutkoski J. Atlin G.. (2019). Enhancing the rate of genetic gain in public-sector plant breeding programs: lessons from the breeder’s equation. Theor. Appl. Genet. 132, 627–645. doi: 10.1007/s00122-019-03317-0 30824972 PMC6439161

[B20] Crestani M. Gonzalez da Silva JoséA. Woyann L. G. Zimmer C. Groli E. Costa de Oliveira A.. (2015). Correlations among industrial traits in oat cultivars grown in different locations of Brazil. Aust. J. Crop Sci. 9, 1182–1189.

[B21] De la Fuente M. C. (2022). “Evaluación de impacto de la variedad Supernova-INIA en la Región de la Araucanía,” in Boletín INIA N° 464, Serie Evaluación de Impacto N° 5 (Santiago, Chile: Instituto de Investigaciones Agropecuarias), 39. p. Available at: https://biblioteca.inia.cl/bitstream/handle/20.500.14001/68616/NR42906.pdf?sequence=7&isAllowed=y (Accessed December 12, 2023).

[B22] Dhanalakshmi T. N. Santosh D. T. Shashidhara N. (2023). “Genetic divergence in plant breeding: forces, markers, and importance for crop Improvement,” in Recent Advances in Agricultural Sciences and Technology. Eds. Biradar N. Shan R. A. Ahmad A. (New Delhi, India: Dilpreet Publishing House), 86–92.

[B23] Estoup A. Jarne P. Cornuet J. M. (2002). Homoplasy and mutation model at microsatellite loci and their consequences for population genetics analysis. Mol. Ecol. 11, 1591–1604. doi: 10.1111/j.1365-294X.2005.02553.x 12207711

[B24] Evanno G. Regnaut S. Goudet J. (2005). Detecting the number of clusters of individuals using the software STRUCTURE: a simulation study. Mol. Ecol. 14 (8), 2611–2620. doi: 10.1111/j.1365-294X.2005.02553.x 15969739

[B25] FAO (2022). FAO Strategy on Climate Change 2022–2031 (Rome: Food and Agriculture Organization of the United Nations), 52. p.

[B26] FDA (2023) Food labeling and nutrition: authorized health claims that meet the significant scientific agreement (SSA) standard, U.S. Food and Drug Administration. Available at: https://www.fda.gov/food/food-labeling-nutrition/authorized-health-claims-meet-significant-scientific-agreement-ssa-standard (Accessed July 19, 2023).

[B27] Fulton T. M. Chunwongse J. Tanksley S. D. (1995). Microprep protocol for extraction of DNA from tomato and other herbaceous plants. Plant Mol. Biol. Rep. 13, 207–209. doi: 10.1007/BF02670897

[B28] Haikka H. Manninen O. Hautsalo J. Pietilä L. Jalli M. Veteläinen M. (2020). Genome-wide association study and genomic prediction for *Fusarium graminearum* resistance traits in Nordic oat (*Avena sativa* L.). Agronomy 10, 174. doi: 10.3390/agronomy10020174

[B29] Havrlentová M. Ondreičková K. Hozlár P. Gregusová V. Mihálik D. Kraic J. (2021). Formation of potential heterotic groups of oat using variation at microsatellite loci. Plants 10, 2462. doi: 10.3390/plants10112462 34834825 PMC8621079

[B30] Henderson C. R. (1976). A simple method for computing the inverse of a numerator relationship matrix used in prediction of breeding values. Biometrics 32 (1), 69–83. doi: 10.2307/2529339

[B31] Informe de expertos (2023) Mercado Latinoamericano de Avena, markert report historical and forecasts market analysis. Available at: https://www.informesdeexpertos.com/informes/mercado-latinoamericano-de-avena (Accessed July 19, 2023).

[B32] Irshad A. Guo H. Zhang S. Liu L. (2020). TILLING in cereal crops for allele expansion and mutation detection by using modern sequencing technologies. Agronomy 10, 405. doi: 10.3390/agronomy10030405

[B33] Jan S. F. Khan M. R. Iqbal A. Khan F. U. Ali S. (2020). Genetic diversity in exotic oat germplasm and resistance against barley yellow dwarf virus. Saudi J. Biol. Sci. 27, 2622–2631. doi: 10.1016/j.sjbs.2020.05.042 32994720 PMC7499088

[B34] Jannink J. L. Gardner S. W. (2005). Expanding the pool of PCR-based markers for oat. Crop Sci. 45, 2383–2387. doi: 10.2135/cropsci2005.0285

[B35] Jennings T. N. Kaus B. J. Mullis T. D. Haig S. M. Cronn R. C. (2011). Multiplexed microsatellite recovery using massively parallel sequencing. Mol. Ecol. Resour. 11, 1060–1067. doi: 10.1038/sj.hdy.6800939 21676207

[B36] Kalisz S. Kramer E. (2008). Variation and constraint in plant evolution and development. Heredity 100, 171–177. doi: 10.1038/sj.hdy.6800939 17268482

[B37] Kassambara A. Mundt F. (2020). Factoextra: extract and visualize the results of multivariate data analyses. R Package Version 1.0.7. Available at: https://CRAN.R-project.org/package=factoextra (Accessed December 12, 2023).

[B38] Kaur G. Kapoor R. Sharma P. Srivastava P. (2021). Molecular characterization of oats (*Avena sativa* L.) germplasm with microsatellite markers. Indian J. Genet. Plant Breed. 81, 144–147. doi: 10.31742/IJGPB.81.1.18

[B39] Khan A. Anjum M. H. Rehman M. K. U. Zaman Q. Ullah R. (2014). Comparative study on quantitative and qualitative characters of different oat (*Avena sativa* L.) genotypes under agro-climatic conditions of Sargodha, Pakistan. Am. J. Plant Sci. 05, 3097–3103. doi: 10.4236/ajps.2014.520326

[B40] Khoury C. K. Brush S. Costich D. E. Curry H. A. Haan S. Engels J. M. M.. (2022). Crop genetic erosion: understanding and responding to loss of crop diversity. New Phytol. 233, 84–118. doi: 10.1111/nph.17733 34515358

[B41] Koide Y. Sakaguchi S. Uchiyama T. Ota Y. Tezuka A. Nagano A. J.. (2019). Genetic properties responsible for the transgressive segregation of days to heading in rice. G3 Genes Genomes Genet. 9, 1655–1662. doi: 10.1534/g3.119.201011 PMC6505171 30894452

[B42] Koroluk A. Paczos-Grzęda E. Sowa S. Boczkowska M. Toporowska J. (2022). Diversity of Polish oat cultivars with a glance at breeding history and perspectives. Agronomy 12, 2423. doi: 10.3390/agronomy12102423

[B43] Koroluk A. Sowa S. Boczkowska M. Paczos-Grzęda E. (2023). Utilizing genomics to characterize the common oat gene pool-the story of more than a century of Polish breeding. Int. J. Mol. Sci. 24 (7), 6547. doi: 10.3390/ijms24076547 37047519 PMC10094864

[B44] Kozak M. Bocianowski J. Liersch A. Tartanus M. Bartkowiak-Broda I. Piotto F. A.. (2011). Genetic divergence is not the same as phenotypic divergence. Mol. Breed. 28, 277–280. doi: 10.1007/s11032-011-9583-9 21841910 PMC3132435

[B45] Krishna A. Ahmed S. Pandey H. C. Bahukhandi D. (2013). Estimates of Genetic variability, heritability and genetic advance of oat (*Avena sativa* L.) genotypes for grain and fodder yield. Agric. Sci. Res. J. 3, 56–61.

[B46] Kumar R. Varghese S. Jayaswal D. Jayaswall K. Yadav K. Mishra G.. (2023). Agro-morphological and genetic variability analysis in oat germplasms with special emphasis on food and feed. PloS One 18, e0280450. doi: 10.1371/journal.pone.0280450 36753474 PMC9907803

[B47] Kumari T. Jindal Y. Singh S. (2017). Estimates of genetic variability, heritability and genetic advance in oats (*Avena* sp.) for seed and fodder yield traits. Forage Res. 43, 110–115.

[B48] Leišová-Svobodová L. Michel S. Tamm I. Chourová M. Janovska D. Grausgruber H. (2019). Diversity and pre-breeding prospects for local adaptation in oat genetic resources. Sustainability 11, 6950. doi: 10.3390/su11246950

[B49] Lê S. Josse J. Husson F. (2008). FactoMineR: An r package for multivariate analysis. J. Stat. Software 25 (1). doi: 10.18637/jss.v025.i01

[B50] Li C. D. Rossnagel B. G. Scoles G. J. (2000). The development of oat microsatellite markers and their use in identifying relationships among *Avena* species and oat cultivars. Theor. Appl. Genet. 101, 1259–1268. doi: 10.1007/s001220051605

[B51] Liu L. Guo H. Zhao L. Wang J. Gu Y. Zhao S. (2009). “Achievements and perspective of crop space breeding in China,” in Induced plant mutation in the genomics era. Ed. Shu Q. Y. (Rome: Food and Agriculture Organization of the United Nations), 213–215.

[B52] Lyubimova A. V. Tobolova G. V. Eremin D. I. Loskutov I. G. (2020). Dynamics of the genetic diversity of oat varieties in the Tyumen region at avenin-coding loci. Vavilov J. Genet. Breed. 24, 123–130. doi: 10.18699/VJ20.607 PMC7716543 33659791

[B53] Mackay I. J. Cockram J. Howell P. Powell W. (2021). Understanding the classics: the unifying concepts of transgressive segregation, inbreeding depression and heterosis and their central relevance for crop breeding. Plant Biotechnol. J. 19, 26–34. doi: 10.1111/pbi.13481 32996672 PMC7769232

[B54] Mahdi T. Mohammad R. (2022). Importance of BLUP method in plant breeding. J. Plant Sci. Phytopathol. 6, 040–042. doi: 10.29328/journal.jpsp.1001072

[B55] Majhi P. (2020). “Heritability and its genetic worth for plant breeding,” in Advances in genetics and plant breeding. Ed. Saidaiah P. (New Delhi, India: Akinik Publications), 69–75.

[B56] Maqbool S. B. Zhong H. Oraby H. F. Sticklen M. B. (2009). “Transformation of oats and its application to improving osmotic stress tolerance,” in Transgenic wheat, barley and oats. Methods in molecular biology. Eds. Jones H. Shewry P. (New Jersey, USA: Humana Press). doi: 10.1007/978-1-59745-379-0_10 19009445

[B57] Mathias-Ramwell M. Salvo-Garrido H. Reyes-Rebolledo M. Montenegro-Barriga A. (2016). Júpiter-INIA: a new oat variety with improved beta-glucan and protein contents. Chil. J. Agric. Res. 76, 401–408. doi: 10.4067/S0718-58392016000400002

[B58] Mazurkievicz G. Ubert I. de P. Krause F. A. Nava I. C. (2019). Phenotypic variation and heritability of heading date in hexaploid oat. Crop Breed. Appl. Biotechnol. 19, 436–443. doi: 10.1590/1984-70332019v19n4a61

[B59] Meira D. Meier C. Olivoto T. Nardino M. Klein L. A. Moro E. D.. (2019). Estimates of genetic parameters between and within black oat populations. Bragantia 78, 43–51. doi: 10.1590/1678-4499.2018116

[B60] Mohanta T. K. Mishra A. K. Mohanta Y. K. Al-Harrasi A. (2021). Space breeding: the next-generation crops. Front. Plant Sci. 12. doi: 10.3389/fpls.2021.771985 PMC8579881 34777452

[B61] Montilla-Bascón G. Sánchez-Martín J. Rispail N. Rubiales D. Mur L. Langdon T.. (2013). Genetic diversity and population structure among oat cultivars and landraces. Plant Mol. Biol. Rep. 31, 1305–1314. doi: 10.1007/s11105-013-0598-8

[B62] Mukaka M. M. (2012). Statistics corner: a guide to appropriate use of correlation coefficient in medical research. Malawi Med. J. 24, 69–71. 23638278 PMC3576830

[B63] Narváez C. Castro M. H. Valenzuela B. J. Hinrichsen R. P. (2001). Patrones genéticos de los cultivares de vides más comúnmente usados en Chile basados en marcadores de microsatélites. Agric. Téc. 61 (3), 249–261. doi: 10.4067/S0365-28072001000300001

[B64] Nava I. C. Duarte I. T. de L. Pacheco M. T. Federizzi L. C. (2010). Genetic control of agronomic traits in an oat population of recombinant lines. Crop Breed. Appl. Biotechnol. 10, 305–311. doi: 10.1590/S1984-70332010000400004

[B65] Nersting L. G. Andersen S. B. von Bothmer R. Gullord M. Jørgensen R. B. (2006). Morphological and molecular diversity of Nordic oat through one hundred years of breeding. Euphytica 150, 327–337. doi: 10.1007/s10681-006-9116-5

[B66] Nouman-Khalid M. Amjad I. Vamuyah-Nyain M. Sulyman-Saleem M. Asif M. Ammar A.. (2021). A Review: TILLING technique strategy for cereal crop development. IJACBS 2 (5), 08–15.

[B67] ODEPA (2023) Estadísticas productivas. Oficina de Estudios y Políticas Agropecuarias. Available at: https://www.odepa.gob.cl/estadisticas-del-sector/estadisticas-productivas (Accessed July 18, 2023).

[B68] Olivoto T. Lúcio A. D. (2020). Metan: An R package for multi-environment trial analysis. Methods Ecol. Evol. 11, 783–789. doi: 10.1111/2041-210X.13384

[B69] Olivoto T. Nardino M. (2021). MGIDI: toward an effective multivariate selection in biological experiments. Bioinformatics 37, 1383–1389. doi: 10.1093/bioinformatics/btaa981 33226063

[B70] Paudel D. Dhungana B. Caffe M. Krishnan P. (2021). A review of health-beneficial properties of oats. Foods 10, 2591. doi: 10.3390/foods10112591 34828872 PMC8625765

[B71] Peakall R. Smouse P. E. (2006). GENALEX 6: Genetic analysis in excel. Population Genet. Software Teach. Res. Mol. Ecol. Notes. 6, 288–295. doi: 10.1111/j.1471-8286.2005.01155.x

[B72] Perry M. C. McIntosh M. S. (1991). Geographical patterns of variation in the USDA soybean germplasm collection: I. morphological traits. Crop Sci. 31, 1350–1355. doi: 10.2135/cropsci1991.0011183X003100050054x

[B73] Premkumar R. Nirmalakumari A. Anandakumar C. R. (2017). Studies on genetic variability and character association among yield and yield attributing traits in oats (*Avena sativa* L.). Int. J. Curr. Microbiol. Appl. Sci. 6, 4075–4083. doi: 10.20546/ijcmas.2017.611.477

[B74] Pritchard J. K. Stephens M. Donnelly P. (2000). Inference of population structure using multilocus genotype data. Genetics 155, 945–959. doi: 10.1093/genetics/155.2.945 10835412 PMC1461096

[B75] Rana M. Gupta S. Kumar N. Ranjan R. Sah R. Gajghate R.. (2019). Genetic architecture and population structure of oat landraces (*Avena sativa* L.) using molecular and morphological descriptors. Indian J. Tradit. Knowl. 18, 439–450.

[B76] Raza Q. Riaz A. Saher H. Bibi A. Raza M. A. Ali S. S.. (2020). Grain Fe and Zn contents linked SSR markers based genetic diversity in rice. PLoS One 15, e0239739. doi: 10.1371/journal.pone.0239739 32986755 PMC7521695

[B77] R Core Team (2022) R: A language and environment for statistical computing. Available at: https://www.R-project.org/.

[B78] Riaz A. Kanwal F. Ahmad I. Ahmad S. Farooq A. Madsen C. K.. (2022). New hope for genome editing in cultivated grasses: CRISPR variants and application. Front. Genet. 13. doi: 10.3389/fgene.2022.866121 PMC9340155 35923689

[B79] Ristaino J. B. Anderson P. K. Bebber D. P. Brauman K. A. Cunniffe N. J. Fedoroff N. V.. (2021). The persistent threat of emerging plant disease pandemics to global food security. Proc. Natl. Acad. Sci. 118, e2022239118. doi: 10.1073/pnas.2022239118 34021073 PMC8201941

[B80] Salgotra R. K. Chauhan B. S. (2023). Genetic diversity, conservation, and utilization of plant genetic resources. Genes 14, 174. doi: 10.3390/genes14010174 36672915 PMC9859222

[B81] Sanchez D. Sadoun S. B. Mary-Huard T. Allier A. Moreau L. Charcosset A. (2023). Improving the use of plant genetic resources to sustain breeding programs’ efficiency. Proc. Natl. Acad. Sci. 120, e2205780119. doi: 10.1073/pnas.2205780119 36972431 PMC10083577

[B82] Šarhanová P. Pfanzelt S. Brandt R. Himmelbach A. Blattner F. (2018). SSR-seq: genotyping of microsatellites using next-generation sequencing reveals higher level of polymorphism as compared to traditional fragment size scoring. Ecol. Evol. 8, 10817–10833. doi: 10.1002/ece3.4533 30519409 PMC6262739

[B83] Silveira S. F. da S. de C. S., Oliveira D. Maltzhan L. E. Corazza T. de V. F. Stulp C.. (2020). Associations between agronomic performance and grain chemical traits in oat. Commun. Plant Sci. 10, 10.26814/cps2020001. doi: 10.26814/cps2020001

[B84] Skendžić S. Zovko M. Živković I. P. Lešić V. Lemić D. (2021). The impact of climate change on agricultural insect pests. Insects 12, 440. doi: 10.3390/insects12050440 34066138 PMC8150874

[B85] Sürek H. Valentine J. (1996). Relationship among some quantitative traits and heritabilities in cultivated oats (*Avena sativa* L.). Tarim Bilim. Derg. 2, 39–43.

[B86] Swarup S. Cargill E. J. Crosby K. Flagel L. Kniskern J. Glenn K. C. (2021). Genetic diversity is indispensable for plant breeding to improve crops. Crop Sci. 61, 839–852. doi: 10.1002/csc2.20377

[B87] Szurman-Zubrzycka M. Kurowska M. Till B. J. Szarejko I. (2023). Is it the end of TILLING era in plant science? Front. Plant Sci. 14. doi: 10.3389/fpls.2023.1160695 PMC10477672 37674734

[B88] Tanhuanpää P. Manninen O. Beattie A. Eckstein P. Scoles G. Rossnagel B.. (2012). An updated doubled haploid oat linkage map and QTL mapping of agronomic and grain quality traits from Canadian field trials. Genome 55, 289–301. doi: 10.1139/g2012-017 22443510

[B89] Tinker N. A. Chao S. Lazo G. R. Oliver R. E. Huang Y. Poland J. A.. (2014). A SNP genotyping array for hexaploid aot. Plant Genome 7, plantgenome2014.03.0010. doi: 10.3835/plantgenome2014.03.0010

[B90] Tinker N. A. Deyl J. K. (2005). A curated internet database of oat pedigrees. Crop Sci. 45, 2269–2272. doi: 10.2135/cropsci2004.0687

[B91] Upadhyaya H. D. Bramel P. J. Ortiz R. Singh S. (2002). Geographical patterns of diversity for morphological and agronomic traits in the groundnut germplasm collection. Euphytica 128, 191–204. doi: 10.1023/A:1020835419262

[B92] Vanjare D. C. Shinde G. C. Shinde S. D. Pawar V. S. (2021). Genetic variability, heritability and genetic advance studies for green forage yield and associated traits in forage oat (*Avena sativa* L.). Int. J. Curr. Microbiol. Appl. Sci. 10, 488–493. doi: 10.20546/ijcmas.2021.1003.064

[B93] Viruel J. Haguenauer A. Juin M. Mirleau F. Bouteiller D. Bouteiller D.. (2018). Advances in genotyping microsatellite markers through sequencing and consequences of scoring methods for *Ceratonia siliqua* (Leguminosae). Appl. Plant Sci. 6 (12), e01201. doi: 10.1002/aps3.1201 30598859 PMC6303155

[B94] Wang L. Xu J. Wang H. Chen T. You E. Bian H.. (2023). Population structure analysis and genome-wide association study of a hexaploid oat landrace and cultivar collection. Front. Plant Sci. 14. doi: 10.3389/fpls.2023.1131751 PMC10070682 37025134

[B95] Weir B. S. (1996). Genetic data analysis II: methods for discrete population genetic data. Sinauer Sunderland Mass, 445. p. 10.1126/science.250.4980.575 17751487

[B96] Wight C. P. Yan W. Fetch J. M. Deyl J. Tinker N. A. (2010). A set of new simple sequence repeat and avenin DNA markers suitable for mapping and fingerprinting studies in oat (*Avena* spp.). Crop Sci. 50, 1207–1218. doi: 10.2135/cropsci2009.09.0474

[B97] Winkler L. R. Michael Bonman J. Chao S. Admassu Yimer B. Bockelman H. Esvelt Klos K. (2016). Population structure and genotype–phenotype associations in a collection of oat landraces and historic cultivars. Front. Plant Sci. 7. doi: 10.3389/fpls.2016.01077 PMC4965477 27524988

[B98] Yan W. Frégeau-Reid J. Pageau D. Martin R. (2016). Genotype-by-environment interaction and trait associations in two genetic populations of oat. Crop Sci. 56, 1136–1145. doi: 10.2135/cropsci2015.11.0678

[B99] Yemataw Z. Blomme G. Muzemil S. Tesfaye K. (2018). Assessing qualitative and phenotypic trait diversity in Ethiopian enset [*Ensete ventricosum* (Welw.) Cheesman] landraces. Fruits 73, 310–327. doi: 10.17660/th2018/73.6.2

[B100] Zadoks J. C. Chang T. T. Konzak C. F. (1974). A decimal code for the growth stages of cereals. Weed Res. 14, 415–421. doi: 10.1111/j.1365-3180.1974.tb01084.x

[B101] Zheng X. Cheng T. Yang L. Xu J. Tang J. Xie K.. (2019). Genetic diversity and DNA fingerprints of three important aquatic vegetables by EST-SSR markers. Sci. Rep. 9, 14074. doi: 10.1038/s41598-019-50569-3 31575997 PMC6773842

[B102] Zimmer C. M. Ubert I. P. Pacheco M. T. Federizzi L. C. (2019). Variable expressivity and heritability of multiflorous spikelets in oat panicles. Exp. Agric. 55, 829–842. doi: 10.1017/S0014479718000418

[B103] Zohary D. Hopf M. (2000). Domestication of plants in the old world: the origin and spread of cultivated plants in West Asia, Europe, and the Nile Valley. 3rd ed (Oxford New York: Oxford University Press).

[B104] Zou M. Tong S. Zou T. Wang X. Wu L. Wang J.. (2023). New method for mutation inducing in rice by using DC electrophoresis bath and its mutagenic effects. Sci. Rep. 13, 6707. doi: 10.1038/s41598-023-33742-7 37185291 PMC10126576

